# Selective depletion of polymorphonuclear myeloid derived suppressor cells in tumor beds with near infrared photoimmunotherapy enhances host immune response

**DOI:** 10.1080/2162402X.2022.2152248

**Published:** 2022-11-30

**Authors:** Takuya Kato, Hiroshi Fukushima, Aki Furusawa, Ryuhei Okada, Hiroaki Wakiyama, Hideyuki Furumoto, Shuhei Okuyama, Seiichiro Takao, Peter L. Choyke, Hisataka Kobayashi

**Affiliations:** Molecular Imaging Branch, Center for Cancer Research, National Cancer Institute, NIH, Bethesda, Maryland, USA

**Keywords:** Myeloid-derived suppressor cell (MDSC), cancer, near-infrared photoimmunotherapy (NIR-PIT), cancer therapy, Ly6G

## Abstract

The immune system is recognized as an important factor in regulating the development, progression, and metastasis of cancer. Myeloid-derived suppressor cells (MDSCs) are a major immune-suppressive cell type by interfering with T cell activation, promoting effector T cell apoptosis, and inducing regulatory T cell expansion. Consequently, reducing or eliminating MDSCs has become a goal of some systemic immunotherapies. However, by systemically reducing MDSCs, unwanted side effects can occur. Near-infrared photoimmunotherapy (NIR-PIT) is a newly developed treatment that selectively kills targeted cells without damaging adjacent normal cells. The aim of this study is to evaluate the antitumor efficacy of MDSC-directed NIR-PIT utilizing anti-Ly6G antibodies to specifically destroy polymorphonuclear (PMN)-MDSCs in the tumor microenvironment (TME) in syngeneic mouse models. PMN-MDSCs were selectively eliminated within tumors by Ly6G-targeted NIR-PIT. There was significant tumor growth suppression and prolonged survival in three treated tumor models. In the early phase after NIR-PIT, dendritic cell maturation/activation and CD8^+^ T cell activation were enhanced in both intratumoral tissues and tumor-draining lymph nodes, and NK cells demonstrated increased expression of cytotoxic molecules. Host immunity remained activated in the TME for at least one week after NIR-PIT. Abscopal effects in bilateral tumor models were observed. Furthermore, the combination of NIR-PIT targeting cancer cells and PMN-MDSCs yielded synergistic effects and demonstrated highly activated host tumor immunity. In conclusion, we demonstrated that selective local PMN-MDSCs depletion by NIR-PIT could be a promising new cancer immunotherapy.

## Introduction

Myeloid-derived suppressor cells (MDSCs) have a well characterized immunosuppressive function and are commonly found in the tumor microenvironment (TME) in many cancers.^1–3^ The presence of MDSCs in tumor tissue is associated with a decrease in mature dendritic cells (DCs) in many murine tumor models.^4,5^ This is because the differentiation of bone marrow progenitor cells into granulocytes, macrophages, and DCs is disturbed by an immunosuppressive TME, resulting from the accumulation of MDSCs. Furthermore, MDSCs are implicated primarily in the suppression of T cells and other cell types in the immune system.^6^ The predominant cytokines and other mediators involved in MDSC-mediated immunosuppression are arginase-1, inducible nitric oxide synthase (iNOS), interleukin (IL)-10, and reactive oxygen specimen among others.^2,6^ These substances prevent T cell activation and promote effector T cell apoptosis.^7^ Furthermore, MDSCs induce regulatory T cell (Treg) expansion in the presence of IFNγ and IL-10, and disrupt innate immunity by interacting with macrophages, NK cells, and NK T cells.^2,8^ Thus, MDSCs act on multiple immune cell populations to promote immunosuppression and tumor progression.

In diseases such as cancer or chronic inflammation, the bone marrow and spleen increase the production of mature and immature myeloid cells comprising a spectrum of cell types from monocytes to neutrophils. Thus, there are two major subsets of MDSCs based on their phenotype and morphology: polymorphonuclear (PMN)-MDSCs representing the neutrophilic end of the spectrum and monocytic (M)-MDSCs representing the monocytic end of the spectrum.^5,9,10^ M-MDSCs suppress T cell responses both in an antigen-specific and antigen-nonspecific manner, while PMN-MDSCs suppress immune responses primarily in an antigen-specific manner. Induction of antigen-specific T-cell tolerance is one of the major hallmarks of these cells.^11,12^ In most solid malignant tumors, PMN-MDSCs were reported to be the predominant subpopulation of MDSCs infiltrating the TME or circulating in the body.^13,14^ In the clinical setting, intratumoral PMN-MDSCs are significantly correlated with poor prognosis in a variety of malignancies.^13,15–17^ Therefore, it could be logical to selectively target intratumoral PMN-MDSCs.

MDSC subpopulations can be distinguished with surface markers such as Ly6G and Ly6C in mice, unlike CD14, CD15, CD66b, and HLA-DR markers used in humans.^10^ Near-infrared photoimmunotherapy (NIR-PIT) is a novel cancer therapy which utilizes antibody-photoabsorber conjugate (APC) and near infrared (NIR) light and induces selective cell death against targeted cells without damaging adjacent normal cells.^18^ With the application of NIR light, rapid cell-specific, necrotic, and highly immunogenic cell death (ICD) is seen in targeted cells.^18–20^ Currently, in Japan, NIR-PIT utilizing human epidermal growth factor receptor (hEGFR) has been used in clinical practice since January 2021.

NIR-PIT was initially developed to target cancer cells, yet it can be applied to other types of cells, such as immunosuppressive cells. For instance, we previously developed NIR-PIT targeting Treg using the surface marker of CD25 or CTLA4, and have shown that intratumoral Tregs are killed, resulting in an antitumor immune activation and tumor growth suppression.^21,22^ Applying the same logic to Ly6G, a surface marker specifically expressed on PMN-MDSCs in mouse models, it was hypothesized that Ly6G-targeted NIR-PIT could selectively deplete PMN-MDSCs thus, activate their host antitumor immunity. Hence, we evaluated the local depletion of PMN-MDSCs in the tumor bed and subsequent antitumor effects of NIR-PIT targeting Ly6G in mouse tumor models.

## Materials and methods

Detailed materials and methods are described in the Supplemental online material.

## Results

### NIR-PIT utilizing Ly6G-IR700 selectively killed PMN-MDSCs

**Figure 1.**
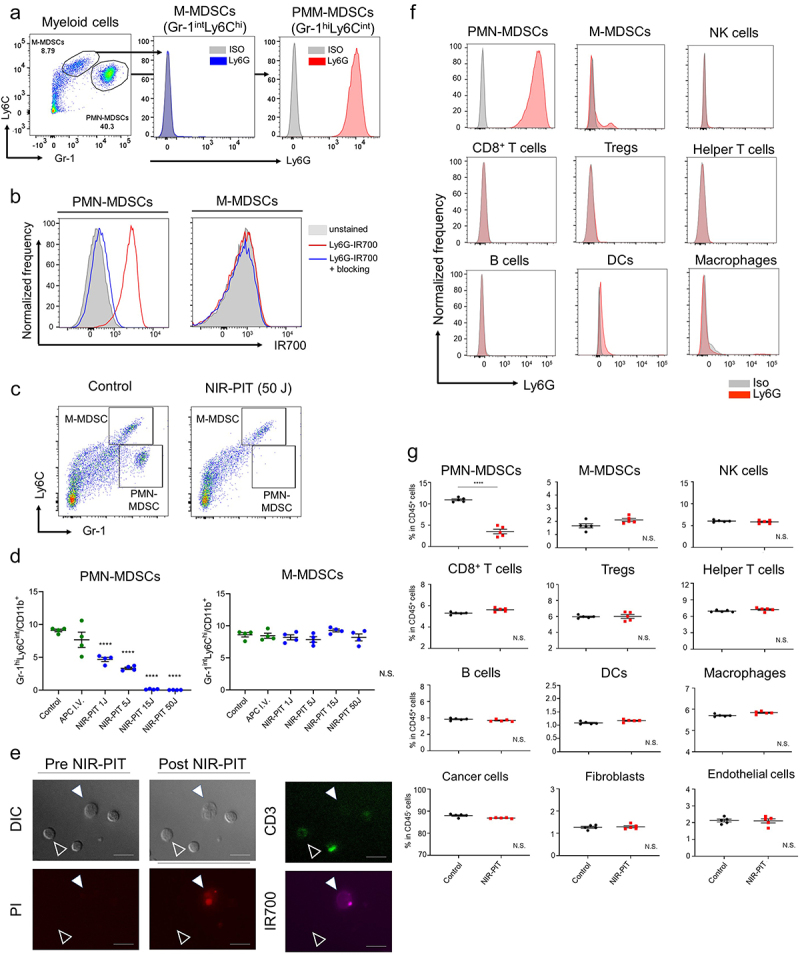
Confirmation of Ly6G expression and *ex vivo* Ly6G-targeted NIR-PIT. (a) PMN-MDSCs were defined as Gr-1^hi^ Ly6C^int^ cells, and M-MDSCs were defined as Gr-1^int^ Ly6C^hi^ cells in CD11b^+^ myeloid cells. (b) The binding of Ly6G-IR700 to PMN- or M-MDSCs. (c and d) The efficacy of Ly6G-targeted NIR-PIT against MDSCs in spleen *ex vivo*. Representative gating strategy and the percentage of PMN- or M-MDSCs in CD45^+^ cells after Ly6G-targeted NIR-PIT *ex vivo* are shown (n = 4; one-way ANOVA followed by Tukey’s test). (e) Microscopic images. Filled arrowhead shows IR700-bound cell, and open arrowhead shows CD3^+^ T cell. Scare bar, 50 µm. (f) Surface expression of Ly6G on various hematopoietic cells. (g) *Ex vivo* selective depletion of PMN-MDSCs within mEERL-hEGFR tumors 1 hour after Ly6G-targeted NIR-PIT (n = 5, unpaired t test). ****, *P* < .0001; N.S., not significant; ISO, isotype control; Ly6G, anti-Ly6G mAb.

The successful conjugation of anti-Ly6G monoclonal antibody (mAb) and IR700 was demonstrated (Figure S1). In this study, we confirmed that Ly6G positive cells could be distinguished using Gr-1 and Ly6C antibodies and defined Gr-1^hi^Ly6C^int^ myeloid cells as PMN-MDSCs and Gr-1^int^Ly6C^hi^ myeloid cells as M-MDSCs (Figure 1(a) and Figure S2). As shown in Figure S3, Gr-1 signal was slightly lower in Ly6G-IR700 i.v. injection only (APC-I.V.) group, suggesting that anti-Gr-1 clone RB6-8C5 may bind the common epitope as Ly6G-IR700 and competed for the binding. However, the signal reduction of Gr-1 was small and did not interfere with the gating on flowcytometric analysis. To verify the *ex vivo* binding of Ly6G-IR700 to PMN- and M-MDSCs, myeloid cells isolated from the spleen were incubated with Ly6G-IR700 (Figure 1(b)). PMN-MDSCs had high IR700 signal, and excess unconjugated anti-Ly6G mAbs neutralized this signal, suggesting that PMN-MDSCs expressed Ly6G, and this binding was specific. However, no signal was observed for M-MDSCs. We quantitatively assessed the cytotoxicity of Ly6G-targeted NIR-PIT against splenocytes (Figure 1(c,d)). PMN-MDSCs were decreased in a NIR light-dose dependent manner. Moreover, the cytotoxicity of NIR-PIT was not seen in M-MDSCs. No direct cytotoxicity against cancer cells was detected (Figure S4). Additionally, microscopic analysis showed that IR700-bound cells were damaged after NIR-PIT, while non-IR700-bound cells had no apparent changes (Figure 1(e)). Next, we assessed the expression of Ly6G and Ly6C in splenocytes (figure 1(f) and Figure S5). Ly6G was expressed on the surface of PMN-MDSCs alone, while Ly6C was expressed on PMN-MDSCs, M-MDSCs, T cells, NK cells, DCs, and macrophages. To confirm which cells in tumors were depleted, *ex vivo* cell viability after Ly6G-targeted NIR-PIT was assessed via flow cytometry (Figure 1(g)). Consistent with Ly6G expression, only PMN-MDSCs were significantly depleted by this treatment. Thus, these results demonstrated that Ly6G-targeted NIR-PIT could selectively destroy PMN-MDSCs without damaging adjacent cells representing other subtypes.

### Distribution of MDSCs and specific binding of Ly6G-IR700 in allograft tumor models

**Figure 2.**
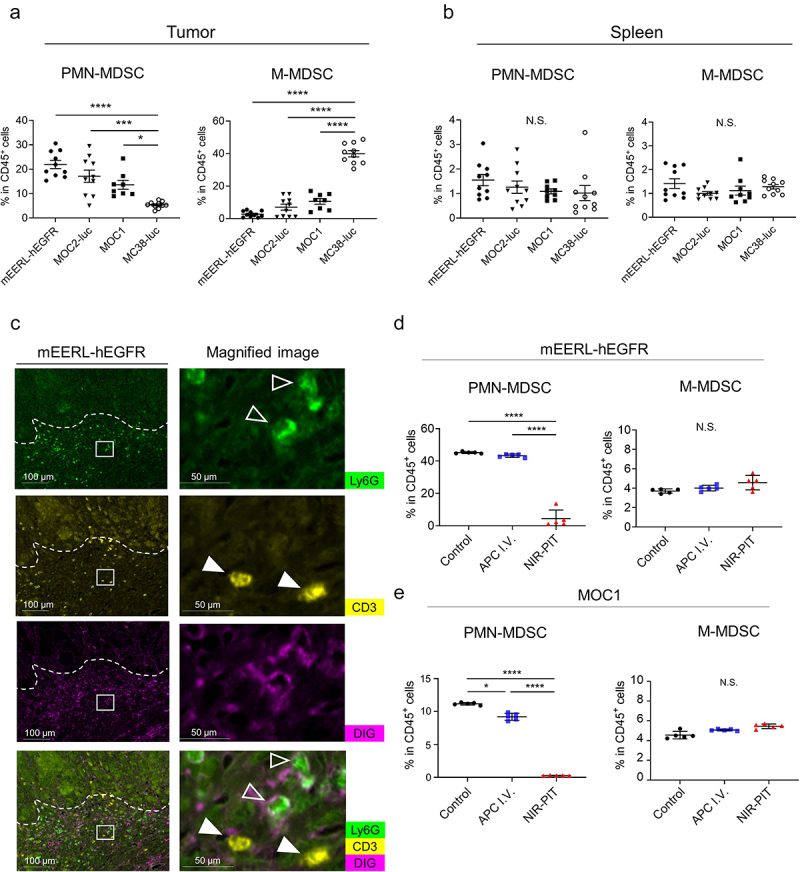
Distribution of MDSCs in various tumors and *in vivo* selective depletion of PMN-MDSCs by Ly6G-targeted NIR-PIT. The percentage of PMN- or M-MDSCs in CD45^+^ cells within tumors (a) or spleens (b) in the four allografts (n = 8–10, means ± SEM; one-way ANOVA followed by Tukey’s test). (c) The distribution of DIG-labeled anti-Ly6G antibody in mEERL-hEGFR tumor detected by multiplex IHC. Ly6G (green), CD3 (yellow), and DIG (magenta). White squares represent the location of the magnified view. Open arrowhead shows Ly6G positive cell; filled arrowhead shows CD3^+^ T cell. (d,e) The percentage of PMN- or M-MDSCs in CD45^+^ cells 1 hour after Ly6G-targeted NIR-PIT *in vivo* models (n = 5; one-way ANOVA followed by Tukey’s test). *, *P*< .05, ***, *P* < .001, ****, *P* < .0001; N.S., not significant.

The number of PMN-MDSCs and M-MDSCs were calculated and compared in four syngeneic tumor models. In mEERL-hEGFR, MOC2-luc, and MOC1 tumors, the number of tumor-infiltrated PMN-MDSCs in CD45^+^ cells were significantly higher compared to MC38-luc tumor, while the number of tumor-infiltrated M-MDSCs was significantly higher in MC38-luc tumors compared to other tumors (Figure 2(a)). In the spleen, no significant differences were found in either MDSC (Figure 2(b)). These results demonstrated that the type and number of tumor infiltrating MDSCs varied by tumor models. In this study, mEERL-hEGFR, MOC2-luc, and MOC1 were used as PMN-MDSCs-rich models and MC38-luc as an M-MDSC-rich model. Next, to evaluate the delivery and specific binding of anti-Ly6G mAbs, multiplex immunohistochemistory (IHC) was performed 1 day after administration of digoxigenin-labeled anti-Ly6G mAb (Ly6G-DIG, Figure 2(c)). Ly6G-DIG was detected on the surface of Ly6G-positive cells, while the DIG signals were not observed on CD3-positive cells. Under the same schedule, *in vivo* Ly6G-targeted NIR-PIT was performed to assess specific cytotoxicity (Figure 2(d,e)). The results showed that only PMN-MDSCs were decreased after NIR-PIT. Thus, Ly6G-IR700 was successfully delivered and bound to target cells within the tumors, and the selective cytotoxicity of Ly6G-targeted NIR-PIT was effective.

### Ly6G-targeted NIR-PIT suppressed tumor growth

**Figure 3.**
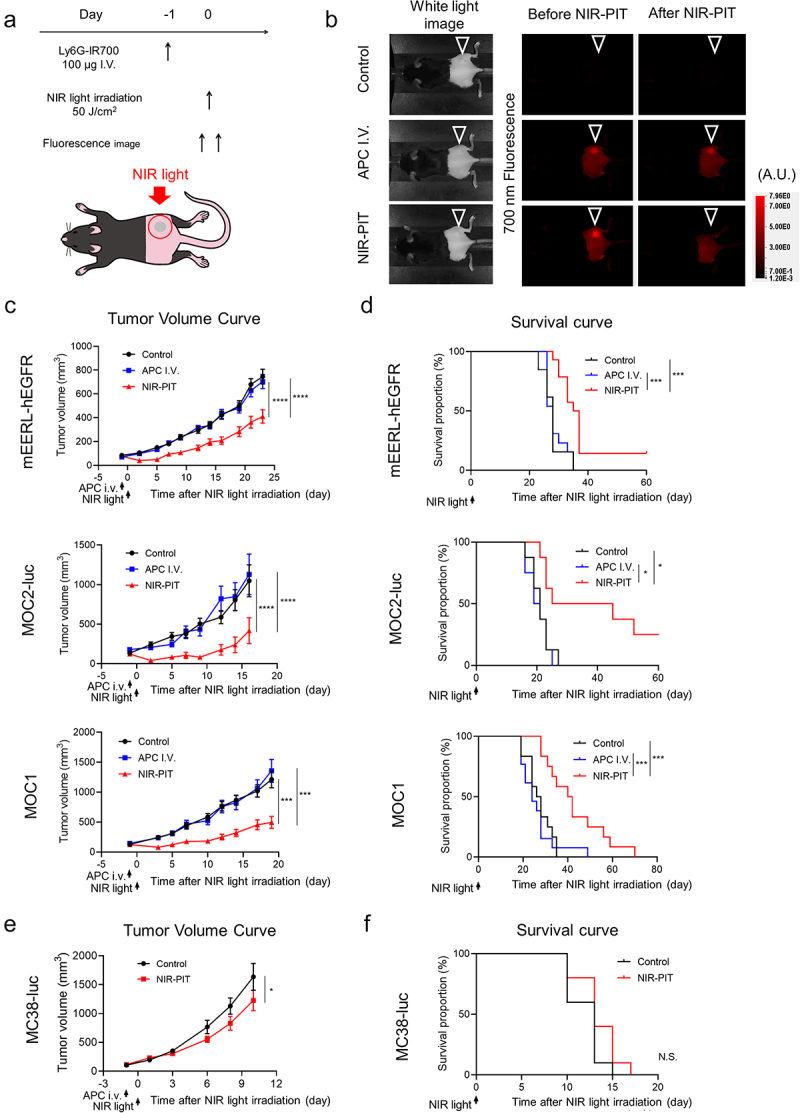
Efficacy of Ly6G-targeted NIR-PIT *in vivo*. (a) Treatment schedule and diagram of NIR light irradiation. The red circle indicates where NIR light was irradiated. (b) Fluorescence images at 700 nm. Arrowheads show tumors. (c) Tumor volume curves for PMN-MDSCs-rich tumors (n = 12–13; mean ± SEM; repeated measures two-way ANOVA followed by Tukey’s test). (d) Survival curves for PMN-MDSCs-rich tumors (n = 12–13; log-rank test with Bonferroni correction). (e) Tumor volume curves for MC38-luc tumors (n = 10; mean ± SEM; repeated measures two-way ANOVA followed by Sidak’s test). (f) Survival curves for MC38-luc tumors (n = 10; log-rank test). *, *P* < .05; ***, *P* < .001; ****, *P* < .0001; N.S., not significant.

We confirmed that the optimal time for NIR light irradiation was approximately 1 day after Ly6G-IR700 administration by the biodistribution of Ly6G-IR700 injection (Figure S6). Next, *in* *vivo* therapeutic effects of Ly6G-targeted NIR-PIT were assessed in the three PMN-MDSC-rich allograft models. The treatment regimen and schema are shown in Figure 3(a). All mice injected with Ly6G-IR700 showed an intense 700 nm fluorescence signal within the tumors, and the signal was attenuated immediately after NIR light irradiation, suggesting the photobleaching of Ly6G-IR700 (Figure 3(b)). In all allograft models, the tumor growth was significantly inhibited in the NIR-PIT group compared with the other two groups (Figure 3(c)). Furthermore, the survival of the NIR-PIT group was also significantly prolonged compared with the other two groups in all allograft models (Figure 3(d)). The efficacy of NIR-PIT against MC38-luc tumor, which contained the least amount of PMN-MDSCs, was also evaluated. Although tumor growth was significantly suppressed, the effect was minimal, and no significant difference in survival was observed (Figure 3(e,f)). Thus, these results demonstrated that Ly6G-targeted NIR-PIT inhibited tumor growth, significantly prolonged survival, and was more effective against PMN-MDSC-rich tumors.

### Anti-tumor host immunity was triggered by Ly6G-targeted NIR-PIT soon after the treatment

**Figure 4.**
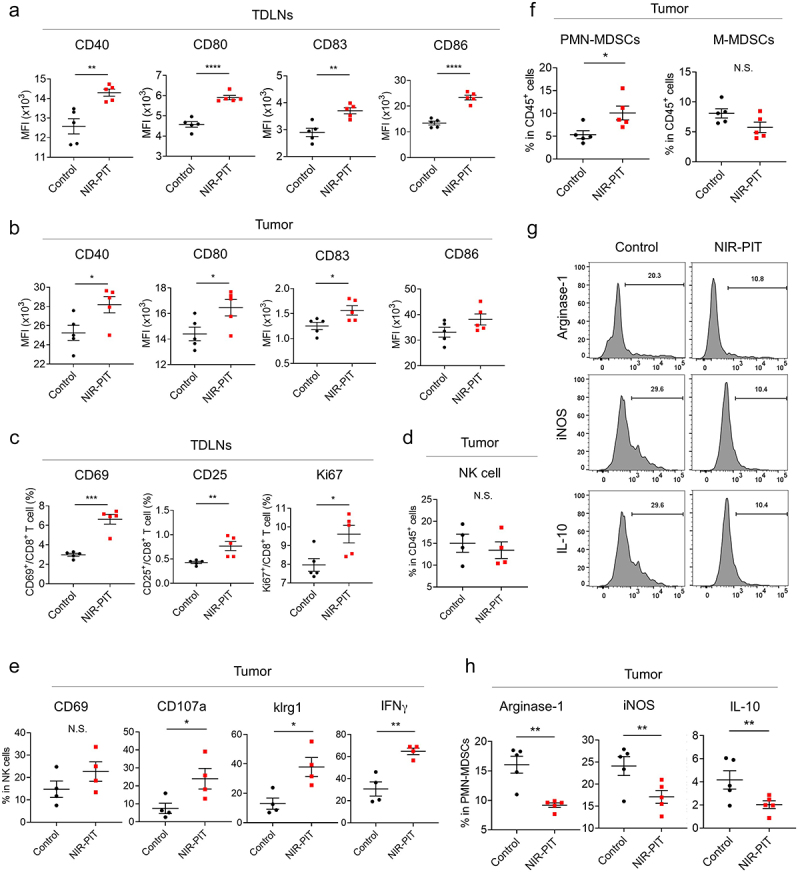
Host immune response after Ly6G-targeted NIR-PIT in early phase. (a-c) Cell populations in the TDLNs and tumors in the mEERL-hEGFR tumors 2 days after Ly6G-targeted NIR-PIT. The expression of CD40, CD80, CD83, and CD86 on DCs in the TDLNs (a) and tumors (b). (c) The expression of CD69, CD25, or Ki67 on CD8^+^ T cell in the TDLNs (a-c, n = 5; mean ± SEM; unpaired *t* test). The population of NK cells (d) and the expression of activated or cytotoxic markers in NK cells (e) 2 days after NIR-PIT (n = 4; mean ± SEM; unpaired *t* test). (f-h) Cell population and intracellular expressions of MDSCs 2 days after NIR-PIT. (f) The cell population of PMN- or M-MDSCs. Representative flow cytometry histogram (g) and quantitative analysis for intracellular expression of arginase-1, iNOS, and IL-10 in PMN-MDSCs (h) were assessed in the intratumoral tissues. (f-h, n = 5; mean ± SEM; unpaired *t* test). *, *P* < .05; **, *P* < .01; ***, *P* < .001; ****, *P* < .0001; N.S., not significant.

First, we confirmed that Ly6G-targeted NIR-PIT did not induce obvious damage to cancer cells in mEERL-hEGFR tumors (Figure S7). Additionally, to test the T-cell dependency of anti-tumor response of Ly6G-targeted NIR-PIT, mEERL-hEGFR tumor growth was evaluated using T-cell deficient athymic mice (Figure S8). No significant difference was observed between the control group and the NIR-PIT group, suggesting the anti-tumor effect of Ly6G-targeted NIR-PIT was T-cell dependent. Next, to assess how the host tumor immunity was stimulated after NIR-PIT, DC maturation and activation in tumors and tumor-draining lymph nodes (TDLNs) were examined 2 days after NIR-PIT. The markers of DC maturation/activation in TDLNs and tumors were significantly increased in the NIR-PIT groups compared to the control groups (Figure 4(a,b)). We also evaluated CD8^+^ T cell activation in TDLNs 2 days after NIR-PIT. Up-regulation of CD69 and CD25 and higher Ki67 positivity were observed in NIR-PIT groups, suggesting CD8^+^ T cells in TDLNs were activated and were proliferating (Figure 4(c)). Although the number of NK cells was not significantly changed, expressions of CD107a and klrg1 and intracellular IFNγ production were increased, suggesting that NK cell activation was promoted (Figure 4(d,e)). Quantitative analyses showed the phenotype of PMN-MDSCs 2 days after NIR-PIT. Although the number of PMN-MDSCs in the tumor was higher in the NIR-PIT group (figure 4(f)), the intracellular expression of arginase-1, iNOS, and IL-10, which indicates T cell suppressive activity, was significantly decreased in the NIR-PIT groups (Figure 4(g,h)). These results suggested that Ly6G-targeted NIR-PIT eliminated highly immunosuppressive PMN-MDSCs perturbing the balance in the TME between immunosuppressive and immune-activating cells.

### Acquired antitumor immunity by NIR-PIT remained energized in the tumor

**Figure 5.**
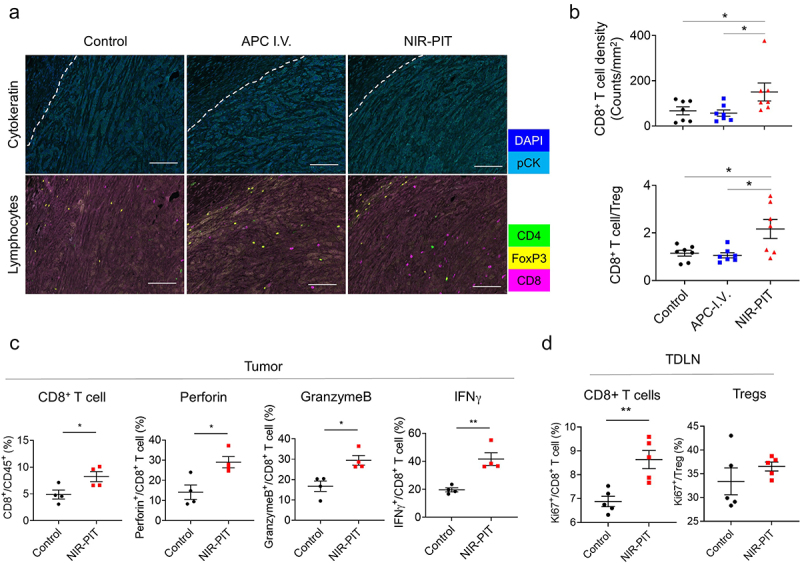
Enhanced antitumor immunity remains 7 days after Ly6G-targeted NIR-PIT. (a) Multiplex IHC images 7 days after Ly6G-targeted NIR-PIT. top, composite images of DAPI (blue) and pCK (cyan) staining; bottom, composite images of lymphocyte markers (CD4, green; Foxp3, yellow; CD8, magenta). White dots line shows the boundary between tumor and stroma. Scare bars, 100 µm. (b) CD8^+^ T cell density and the ratio of CD8^+^ T cell to Treg within the tumor quantified from IHC images (n = 7; one-way ANOVA followed by Tukey’s test). (c) The population of CD8^+^ T cell and the expression of cytotoxic markers in CD8^+^ T cells were evaluated 7 days after the therapy via flow cytometry (n = 4; mean ± SEM; unpaired *t* test). (d) The expression of Ki67 in CD8^+^ T cells and Tregs was evaluated in the TDLNs 7 days after NIR-PIT (n = 5; mean ± SEM; unpaired *t* test). *, *P* < .05; **, *P*< .01.

We next assessed whether the balance between CD8^+^ T cells and Tregs was improved by Ly6G-targeted NIR-PIT. Tumors were harvested 7 days after each treatment, and tumor-infiltrating lymphocytes (TILs) were analyzed by multiplex IHC (Figure 5(a)). The quantification of CD8^+^ T cell density was significantly higher in the NIR-PIT group than in the other two groups (Figure 5(b)). Furthermore, the ratio of CD8^+^ T cells to Tregs, which is a well-known index of strong antitumor immunity,^23^ was significantly higher compared with other groups. Additionally, we further assessed the cytotoxic potential of CD8^+^ T cells in the tumor 7 days after NIR-PIT. In the NIR-PIT group, not only was the number of CD8^+^ T cells increased but they displayed elevated expression of cytotoxic markers, including IFNγ, Perforin, and GranzymeB (Figure 5(c)). Also in TDLNs, Ki67 expression in CD8^+^ T cells remained at higher levels, while no significant difference in Tregs was observed (Figure 5(d)). Thus, these results confirmed that Ly6G-targeted NIR-PIT elicited a potent T cell-mediated antitumor immune reaction.

### Abscopal effects of PMN-MDSC-targeted NIR-PIT

**Figure 6.**
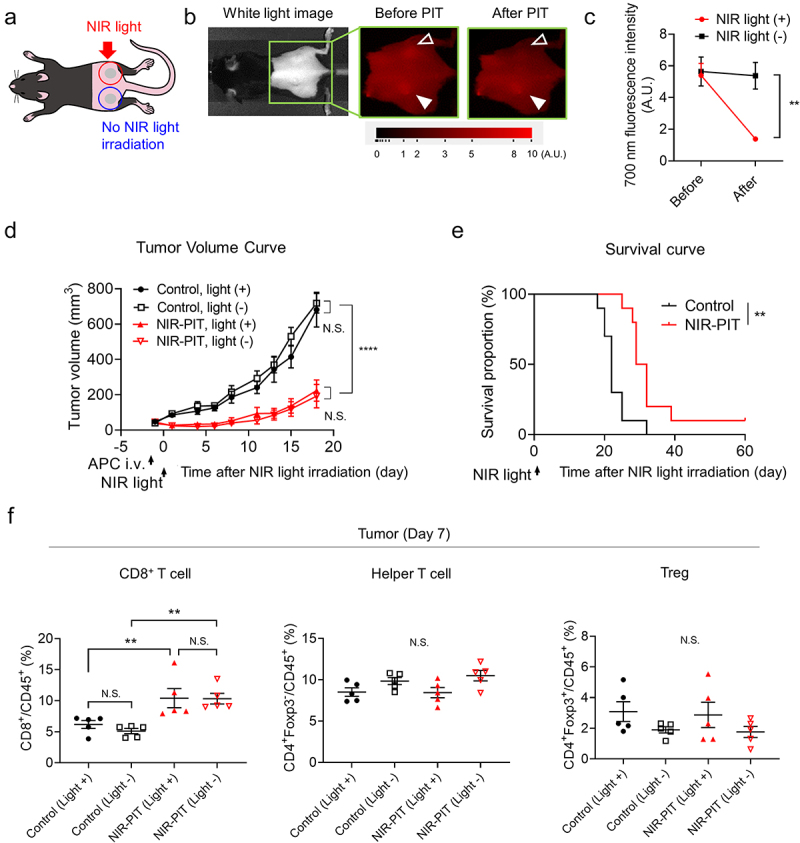
*In vivo* efficacy of Ly6G-targeted NIR-PIT in a bilateral tumor model. (a) Diagram of NIR light irradiation. The red circle indicates where NIR light was irradiated, and the blue circle shows a contralateral tumor shielded from NIR light. (b) Fluorescent images of 700 nm. Open arrowheads show tumors irradiated with NIR light, filled arrowhead shows tumors without NIR light irradiation. (c) Quantification of fluorescence intensity in the tumors. (n = 10; paired *t* test). (d) Tumor volume curves (n = 10; mean ± SEM; repeated measures two-way ANOVA followed by Tukey’s test). (e) Survival curves (n = 10; log-rank test). (f) T cell population in CD45^+^ cells in the tumors 7 days after NIR-PIT (n = 5; mean ± SEM; one-way ANOVA followed by Tukey’s test). **, *P* < .01; ****, *P*< .0001; N.S., not significant.

To evaluate the systemic antitumor immunity induced by NIR-PIT, Ly6G-targeted NIR-PIT was performed unilaterally in a bilateral mEERL-hEGFR tumor model. The treatment schema is shown in Figure 6(a). After NIR light administration, the 700 nm fluorescence signal was significantly decreased in the NIR light irradiated side tumor, while it was unchanged in the contralateral tumor (Figure 6(b,c)). However, tumor growth was significantly suppressed not only in the treated tumors but also in untreated tumors (Figure 6(d)). Also, animals in the NIR-PIT group demonstrated significantly prolonged survival compared to the control group (Figure 6(e)). We then evaluated TILs in the tumors 7 days after unilateral Ly6G-targeted NIR-PIT. The number of CD8^+^ T cells in the untreated and treated tumors after NIR-PIT was significantly higher than in the control groups (figure 6(f)). No significant changes in helper T cells and Tregs were observed.

### Immunogenic memory acquired after Ly6G-targeted NIR-PIT

To test for immune memory, mice with mEERL-hEGFR tumors that had undergone Ly6G-targeted NIR-PIT were re-inoculated with mEERL-hEGFR cells on the contralateral dorsum approximately 12 weeks after the initial NIR-PIT (Figure S9). All mice in the re-inoculation group rejected the newly injected mEERL-hEGFR cancer cells definitively. This result indicated the acquisition of an anti-tumor immune memory after Ly6G-targeted NIR-PIT.

### Simultaneous NIR-PIT-targeting of cancer cells and PMN-MDSCs resulted in more effective tumor suppression than targeting either alone

**Figure 7.**
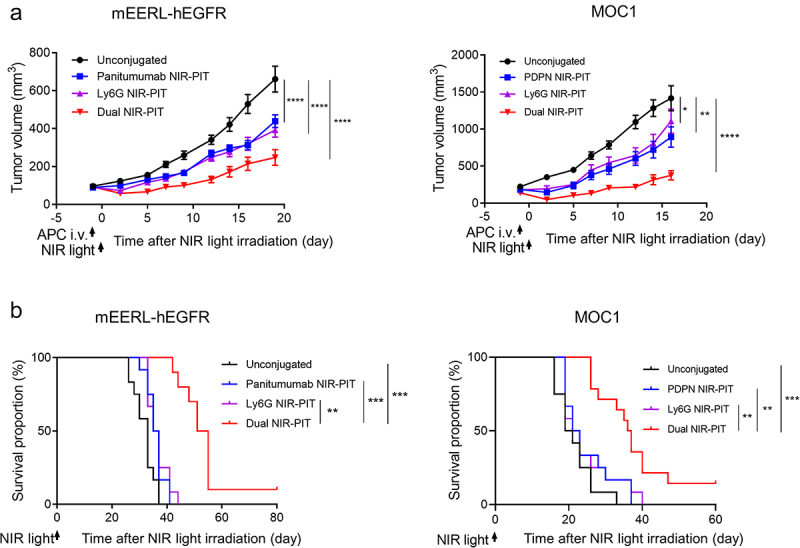
Efficacy of combined NIR-PIT targeting PMN-MDSCs and cancer cells. (a) Tumor volume curves. (n = 12–13; mean ± SEM; repeated measures two-way ANOVA followed by Tukey’s test). (b) Survival curves (n = 12–13; log-rank test with Bonferroni correction). *, *P* < .05; **, *P* < .01; ***, *P* < .001; ****, *P* < .0001.

To evaluate the synergistic effect of NIR-PIT targeting cancer cells and PMN-MDSCs, we performed dual-targeted NIR-PIT in two allograft models. As a cancer cell-targeting agent, panitumumab was used for mEERL-hEGFR tumors, and anti- PDPN mAb was used for MOC1 tumors. The treatment schedule is shown in Figure S10. The tumor growth in mono-targeted NIR-PIT was suppressed compared with the control group; however, the dual NIR-PIT group inhibited tumor progression more effectively in both allograft models (Figure 7(a)). Furthermore, the survival of animals in the dual NIR-PIT group was significantly prolonged compared to other groups (Figure 7(b)). Therefore, we concluded that Ly6G-targeted NIR-PIT had a synergistic effect on cancer cell-targeted NIR-PIT to suppress tumor growth.

## Discussion

The immunosuppressive role played by MDSCs has made them a target for several therapies. Low-dose gemcitabine and 5-fluorouracil are known to reduce MDSCs in tumors.^3,24,25^ However, these treatments also damage other cells, including effector cells in the immune system, thus, causing counterproductive effects.^26^ New therapeutic approaches using all-trans retinoic acid, 1-methyltryptophan, colony stimulating factor-1 receptor, and phosphodiesterase-5 (PDE-5) inhibitor have been considered as MDSC-targeted treatment but are still under investigation and have not been deployed clinically.^26–30^ In this study, we demonstrated that Ly6G-targeted NIR-PIT could trigger an anti-tumor effect by local elimination of PMN-MDSCs within the tumor. Unlike systemic depletion by continuous antibody administration, Ly6G-targeted NIR-PIT does not affect adjacent normal cells or uninvolved organs outside the NIR light-irradiated area and, therefore, would preserve the host immune system. The depletion of PMN-MDSC by Ly6G-IR700 injection alone was not substantial enough to exert a therapeutic effect because the dose of Ly6G-IR700 used in this study was relatively small and administrated as a single injection.^31^ Thus, we propose Ly6G-targeted NIR-PIT as a new therapeutic approach to deplete PMN-MDSCs locally.

This study showed that host-acquired immunity was activated after Ly6G-targeted NIR-PIT. It is well-known that PMN-MDSCs suppress T cell proliferation while promoting antigen-specific T cell suppression/tolerance and T cell apoptosis, resulting in suppressed tumor immunity.^9,14^ Based on this finding, NIR-PIT was used to selectively kill MDSCs, resulting in almost completely MDSC reduction of pre-treatment. Despite this treatment, PMN-MDSC counts 2 days after NIR-PIT doubled compared to pre-treatment. However, the percentage of immunosuppressive MDSCs, such as arginase-1 positive cells, decreased. This is probably because less mature MDSC populations migrated into the TME after inhibitory PMN-MDSCs were selectively depleted by Ly6G-targeted NIR-PIT. Depletion of PMN-MDSCs triggered both innate and acquired immune responses, resulting in the migration of highly cytotoxic CD8^+^ T cells into the tumor. Meanwhile, the results of the bilateral tumor experiment in which only one tumor was treated, but the contralateral tumor also responded, suggested that the activation of the immune system was systemic and not limited to the treated tumor.

There is considerable variability in the distribution and subtypes of MDSCs within cancers. This can be seen even among the models used in this study. Unsurprisingly, the PMN-MDSC-dominant tumors responded well to Ly6G-targeted NIR-PIT, while M-MDSC-dominant tumors were much less responsive. In the latter case, targeting Ly6C for NIR-PIT might be more effective. Similar restrictions apply to previously developed Treg-targeted NIR-PIT which was effective in Treg-rich environments but limited in effectiveness in poorly immunogenic tumors because of the smaller number of Tregs.^32,33^ In MDSC-dominant tumors with smaller Treg content, Ly6G-targeted NIR-PIT is predicted to be more valuable as NIR-PIT targeting immune cells rather than NIR-PIT targeting Tregs (e.g., NIR-PIT targeting Tregs had minimal effect for mEERL-hEGFR tumors).^33^ Therefore, when contemplating future uses of this treatment, the selection of the most appropriate NIR-PIT targeting immune cells (PMN-MDSC or Treg) rests with the determination of the relative dominance of these cell types in the TME.

There are some limitations in this study. First, Ly6G and Ly6C are murine-specific surface antigens used for distinguishing MDSCs and may not be relevant to human MDSCs. In humans, several surface markers such as CD15, LOX-1, and S100A9 have been used to refine the identification of PMN-MDSCs and M-MDSCs.^14,34^ Additionally, the C-X-C chemokine receptor 2 (CXCR2) is expressed on MDSCs, and the CXCR2-axis plays an essential role in the migration of immunosuppressed MDSCs into the TME.^35^ These molecules may be alternative candidates to Ly6G-targeted NIR-PIT to eliminate PMN-MDSCs in the context of human tumors, but further investigation will be required. Second, Ly6G-expressing myeloid cells were defined as PMN-MDSCs. We could not exclude the possibility that Ly6G-positive neutrophils were admixed with other myeloid cells despite being classified as PMN-MDSCs. Furthermore, orthotopic models may be more appropriate to evaluate the immune response by NIR-PIT because of organ-specific TME changes.^36^

In conclusion, we demonstrated that local PMN-MDSC depletion in the tumor using Ly6G-targeted NIR-PIT enhanced the host antitumor immune system, resulting in suppression of tumor progression not only in directly irradiated tumors but also in non-irradiated tumors in the same animal. This systemic or abscopal effect was augmented when combined with cancer cell-targeted NIR-PIT. Finally, NIR-PIT targeting PMN-MDSCs may be effective in poorly immunogenic tumors that are difficult to treat by Treg-targeted NIR-PIT and will expand the number of applications of immunosuppressive cell-targeted NIR-PIT.

## Supplementary Material

Supplemental Material
