# Supplementary material for: Selective depletion of polymorphonuclear myeloid derived suppressor cells in tumor beds with near infrared photoimmunotherapy enhances host immune response
Source: Oncoimmunology. 2022 Nov 30;11(1):2152248. doi: 10.1080/2162402X.2022.2152248 (PMC9718564; doi:10.1080/2162402X.2022.2152248)
Supplement: Supplemental Material [file KONI_A_2152248_SM6652.docx]

Supplemental online material for

**Selective Depletion of Polymorphonuclear Myeloid Derived Suppressor Cells in Tumor Beds with Near Infrared Photoimmunotherapy Enhances Host Immune Response**

**Authors:** Takuya Kato^1^, Hiroshi Fukushima^1^, Aki Furusawa^1^, Ryuhei Okada^1^, Hiroaki Wakiyama^1^, Hideyuki Furumoto^1^, Shuhei Okuyama^1^, Seiichiro Takao^1^, Peter L. Choyke^1^, Hisataka Kobayashi^1^

**Affiliations:**

^1^Molecular Imaging Branch, Center for Cancer Research, National Cancer Institute, NIH, Bethesda, Maryland 20892-1088, United States

*Corresponding author. Hisataka Kobayashi, M.D., Ph.D.

Molecular Imaging Branch, Center for Cancer Research, National Cancer Institute, NIH, 10 Center Drive, Bethesda, MD, 20892, USA

Tel: 240-858-3069; Fax: 240-541-4527; E-mail: kobayash@mail.nih.gov

**List of** **Supplemental online material**

1. **Abbreviations**
2. **Supplemental materials and methods.**

- Reagents
- Synthesis of antibody-IR700 conjugate
- Cell culture
- Animal and tumor model
- Flow cytometric analysis of Ly6G-IR700 binding
- *Ex vivo* NIR-PIT
- *In vivo* NIR-PIT
- Multiplex immunohistochemistry (IHC)
- DIG-labeled antibody detection by IHC
- Flow cytometric analysis
- Statistical analysis

1. **Supplementary Figures**

Supplementary Figure S1. Verification of the conjugation of anti-Ly6G-mAb to IR700.

Supplementary Figure S2. Gating strategy and evaluation of Ly6G expressions for MDSCs using Gr-1 and Ly6C antibodies.

Supplementary Figure S3. Gating examples with Gr-1 and Ly6G-IR700.

Supplementary Figure S4. The efficacy of Ly6G-targeted NIR-PIT against cancer cells.

Supplementary Figure S5. Ly6C expression on various hematopoietic cells in spleen.

Supplementary Figure S6. In vivo IR700 fluorescence imaging of mEERL-hEGFR and MOC1 tumor.

Supplementary Figure S7. Histological changes after Ly6G-targeted NIR-PIT in mEERL-hEGFR tumor bearing mice.

Supplementary Figure S8. The efficacy of in vivo Ly6G-targeted NIR-PIT in athymic mice.

Supplementary Figure S9. Re-inoculation of cancer cells after Ly6G-targeted NIR-PIT.

Supplementary Figure S10. Treatment regimen and 700 nm fluorescence images for combined NIR-PIT.

**1.** **Abbreviations**

near-infrared photoimmunotherapy (NIR-PIT), myeloid-derived suppressor cell (MDSC), polymorphonuclear-MDSC (PMN-MDSC), monocytic-MDSC (M-MDSC), monoclonal antibody (mAb), IRDye700DX (IR700), antibody-photoabsorber conjugate (APC), podoplanin (PDPN), tumor microenvironment (TME), regulatory T cell (Treg), epidermal growth factor receptor (EGFR), dendritic cell (DC), nitric oxide (NO) , inducible nitric oxide synthase (iNOS), interleukin (IL), interferon (IFNγ), NK cell (natural killer cell), immunogenic cell death (ICD), cytotoxic T lymphocyte associated protein 4 (CTLA4), fetal bovine serum (FBS), penicillin/streptomycin (P/S), immunohistochemistry (IHC), size-exclusion chromatography (SEC), propidium iodide (PI), digoxigenin (DIG), bioluminescence images (BLI), tumor infiltrating lymphocyte (TIL), pan-cytokeratin (pCK), tumor draining lymph node (TDLN), phosphodiesterase-5 (PDE-5), C-X-C chemokine receptor 2 (CXCR2)

**2. Supplemental materials and methods.**

**Reagents**

We purchased IRDye700DX NHS ester (IR700), a water-soluble silica-phthalocyanine derivative from LI-COR Bioscience (Lincoln, NE, USA). Panitumumab, a fully humanized IgG2 monoclonal antibody (mAb) targeting hEGFR, was purchased from Amgen (Thousand Oaks, CA, USA). Anti-mouse Ly6G (clone 1A8) and anti-mouse podoplanin (PDPN, clone 8.1.1) mAbs were purchased from Bio X Cell (Lebanon, NH, USA). All other chemicals were of reagent grade.

**Synthesis of antibody-IR700 conjugate**

IR700 was conjugated with mAbs in accordance with prior reports [26]. Briefly, 1 mg of mAb was incubated for 1 hour at room temperature with a 5-fold molar excess of IR700 (10 mmol/L in DMSO) in 0.1 mol/L Na_2_HPO_4_ (pH 8.5). A filter column (Sephadex G 25 column, PD-10: GE Healthcare, Piscataway, NJ, USA) was used to purify the mixture. We abbreviate APCs as follows; panitumumab-IR700 as pan-IR700, anti-Ly6G mAb-IR700 as Ly6G-IR700, and anti-PDPN mAb-IR700 as PDPN-IR700, respectively.

**Cell culture**

hEGFR-expressing murine oropharyngeal cell line (mEERL-hEGFR) was a kind gift from Dr. Chad Spanos, Sanford Research [27,28]. MOC1 and MOC2 cells (murine oral cancers) were purchased from Kerafast (Boston, MA, USA). MC38 (colon cancer, kind gift from Claudia Palena, NCI/NIH, MD, USA, 2015) and MOC2 cells stably expressing luciferase (MC38-luc and MOC2-luc, generated via stable transduction with RediFect Red-Fluc lentivirus from PerkinElmer) were used in this study. mEERL-hEGFR cells were cultured in DMEM/F-12 medium (Thermo Fisher Scientific, Waltham, MA, USA) supplemented with 10% fetal bovine serum (FBS, Thermo Fisher Scientific), 100 I.U./mL penicillin/streptomycin (P/S, Thermo Fisher Scientific), and 1 × human keratinocyte growth supplement (Thermo Fisher Scientific) in a humidified incubator at 37 °C in an atmosphere of 5% CO_2_ as previously reported [28]. MC38-luc cells were cultured in RPMI1640 supplemented with 10% FBS and 100 I.U./mL P/S. MOC1 and MOC2-luc cells were cultured in the mixture of IMDM medium and Ham’s Nutrient Mixture F12 Media (at a ratio of 2:1, GE Health Healthcare Life Sciences) supplemented with 5% FBS, 100 I.U./mL P/S, 5 ng/mL insulin (MilliporeSigma Burlington, MA, USA), 40 ng/mL hydrocortisone (MilliporeSigma), and 3.5 ng/mL human recombinant EGF (MilliporeSigma). Cells were authenticated via *in vitro* growth characteristics. Identification of cell lines was evaluated via short tandem repeat (STR) profiling. For MC38-luc, MOC1, and MOC2-luc cells, the matching score was above 80% indicating the cell line identity was authentic (June 2021, IDEXX Bioanalytics). For mEERL-hEGFR, STR profile did not match any known cell lines (April 2021, ATCC). Mycoplasma testing was performed by PCR for MC38-luc, MOC1, and MOC2-luc (IDEXX Bioanalytics), and by MycoAlert PLUS Mycoplasma Detection Kit (Lonza) for mEERL-hEGFR. All the cell lines were negative.

**Animal and tumor model**

All procedures were in compliance with the Guide for the Care and Use of Laboratory Animals and approved by the local Animal Care and Use Committee (MIP-003-4-E, project number P214396). Six- to eight-week-old female C57BL/6 mice (strain #000664) were purchased from The Jackson Laboratory (Bar Harbor, ME, USA). The dorsal lower body was shaved before NIR light irradiation and image analysis. Tumors were established via subcutaneous injection of 1 × 10^6^ cells for mEERL-hEGFR, MOC2-luc, and MC38-luc tumors or 3 × 10^6^ cells for MOC1 tumor in the right or both sides of dorsal flank for each model. Mice with tumors reaching approximately 50-100 mm^3^ in volume were randomized for the experiments. Mice were monitored each day, and tumor volume (length × width^2^ × 0.5) was measured three times a week until the mEERL-hEGFR tumor volume reached 1,000 mm^3^ (2,000 mm^3^ for other tumors), then the mice were euthanized with CO_2_ inhalation. In the bilateral model, mice were euthanized when either tumor reached its endpoint. Tumor disappearance for 4 weeks or longer after treatment was defined as complete remission.

**Flow cytometric analysis of Ly6G-IR700 binding**

MDSCs were isolated from the spleen of mEERL-hEGFR tumor-bearing mouse using EasySep™ Mouse MDSC (CD11b^+^Gr-1^+^) Isolation Kit (StemCell Technologies Inc, Vancouver, Canada). 1 × 10^6^ isolated cells were incubated with Ly6G-IR700 (10 μg/mL) for 1 hour at 37 °C. After washing with PBS, the stained cells were analyzed by flow cytometry (FACSLyric, BD Biosciences) and Flowjo software (BD Biosciences).

***Ex vivo* NIR-PIT**

Spleens and tumors in mEERL-hEGFR-tumor bearing mice were harvested, and single cell suspensions were performed. Red blood cells (RBC) were removed by incubating with RBC lysis buffer (BioLegend, San Diego, CA, USA). For examining cytotoxicity by Ly6G-targeted NIR-PIT, 3 million splenocytes or tumor cells were incubated with 10 μg/mL of Ly6G-IR700 for 1 hour at 4 °C. After washing the cells with PBS, NIR light (690 nm, 150 mW/cm^2^) was irradiated on splenocytes at 0 to 50 J/cm^2^. Tumor cells were irradiated with NIR light at 50 J/cm^2^. After 15 minutes, cells were stained with antibodies and analyzed by flow cytometry. To evaluate morphological changes by NIR-PIT, 1 × 10^4^ single suspended splenocytes were seeded into a glass-bottomed 35 mm dish. Incubated with Ly6G-IR700 and anti-CD3 antibody (clone 145–2C11, BioLegend) for 1 hour at 37 °C, then, the cells were irradiated with NIR light. 1 µg/mL propidium iodide (PI, MilliporeSigma) was added just before NIR light irradiation. Transmitted light differential interference contrast (DIC) images and fluorescent images were obtained before and 15 minutes after the light irradiation with a microscope (IX81; Olympus America, Melville, NY, USA).

***In vivo* NIR-PIT**

For Ly6G-targeted NIR-PIT experiments, tumor-bearing mice were randomized into two or three groups as follows: (i) no treatment (control), (ii) intravenous injection of Ly6G-IR700 (100 μg) without NIR light irradiation (APC-I.V.), (iii) intravenous injection of Ly6G-IR700 (100 μg) followed by NIR light irradiation (NIR-PIT). 24 hours after Ly6G-IR700 administration, NIR light (690 nm, 150 mW/cm^2^, 50 J/cm^2^) was applied to the tumors. The mouse was wrapped in aluminum foil with a portal only for the target tumor; other tumors were shielded from NIR light exposure. The mice with complete response after NIR-PIT in mEERL-hEGFR tumors were re-inoculated with same cancer cells in the contralateral flank. In the bilateral model, only right-sided tumors were irradiated with NIR light at 50 J/cm^2^ while the left-sided tumors were protected from NIR light by foil. In combined NIR-PIT experiments, intravenous injection of mixed antibody/APC followed by NIR light irradiation were performed as follows: i) mixed 50 μg panitumumab or anti-PDPN mAb and 50 μg anti-Ly6G-mAb (I.V. group), ii) mixed 50 μg pan-IR700 or PDPN-IR700 and 50 μg anti-Ly6G-mAb (Panitumumab or PDPN NIR-PIT group), iii) mixed 50 μg panitumumab or anti-PDPN mAb and 50 μg Ly6G-IR700 (Ly6G NIR-PIT group), and iv) mixed 50 μg pan-IR700 or PDPN-IR700 and 50 μg Ly6G-IR700 (Dual NIR-PIT group). 24 hours after administration, NIR light was applied to tumors at 50 J/cm^2^ on day 0 in all groups. Before and after NIR-PIT, 700 nm fluorescent images with white light pictures were obtained (Pearl Imager, LI-COR Biosciences).

**Multiplex immunohistochemistry (IHC)**

Multiplex IHC was carried out as previously described using Opal Automation IHC Kit (Akoya Bioscience) and Bond RXm autostainer (Leica Biosystems) [29]. The following antibodies and DAPI were used: anti-pan cytokeratin (pCK, rabbit poly, Bioss), anti-CD3 (rabbit poly, Thermo Fisher Scientific), anti-CD8 (clone EPR20305, Abcam), anti-CD4 (clone EPR19514, Abcam), anti-FoxP3 (clone 1054C, Novus Biologicals), anti-Ly6G (clone E6Z1T, Cell Signaling), anti-Ly6C (rabbit poly, Invitrogen), anti-Granzyme B (rabbit poly, Abcam), and anti-digoxigenin (DIG, clone 9H27L19, Thermo Fisher Scientific) antibodies. Coverslips were covered with a drop of ProLong Diamond Antifade Mountant (Thermo Fisher Scientific). Staining slides were imaged with Mantra Quantitative Pathology Workstation (Akoya Biosystems).

**DIG-labeled antibody detection by IHC**

Anti-Ly6G mAb (1 mg, 6.8 nmol) and DIG succinimidyl ester (50 μg, Thermo Fisher Scientific) were used for labeling of antibodies with DIG. We abbreviated DIG-labeled anti-Ly6G antibodies as Ly6G-DIG. 100 μg of Ly6G-DIG was injected via tail vein into mEERL-hEGFR tumor-bearing mouse. The tumors were extracted 1 day after Ly6G-DIG administration, then fixed with 10% formalin, embedded in paraffin and thinly sliced. The distribution of Ly6G-DIG was detected by anti-DIG staining.

**Flow cytometric analysis**

To evaluate expression of Ly6G and Ly6C, four tumor bearing-mice (mEERL-hEGFR, MOC1, MOC2-luc, and MC38-luc tumors) were euthanized when established tumor volume reached approximately 150 mm^3^. When observing cytotoxicity or host tumor immunity by Ly6G-targeted NIR-PIT *in vivo*, tumors and tumor draining lymph nodes (TDLNs) were harvested at the designated time (1 day, 2 days, and 7 days after Ly6G-targeted NIR-PIT). Single-cell suspension was obtained as previously described [30]. The cells were stained with the following antibodies: anti-CD3e (clone 145–2C11), anti-CD4 (clone RM4-5), anti-CD11b (clone M1/70) anti-CD11c (N418), anti-CD19 (6D5), anti-CD25 (PC61), anti-CD31 (390), anti-CD45 (30-F11), anti-CD69 (H1.2F3), anti-CD86 (GL-1), anti-CD107 (LAMP-1, 1D413), anti-CD140a (APA5), anti-CD326 (EpCAM, G8.8), anti-F4/80 (BM8), anti-Gr-1 (RB6-8C5), anti-IL-10 (JES5-16E3), anti-Ki67 (10A8), anti-Ly6C (HK1.4), anti-Ly6G (1A8), anti-I-A/I-E (M5/114.15.2 ), anti-NK1.1 (PK136), anti-rat IgG2aκ (RTK2758), anti-rat IgG2cκ (RKT4174) were obtained from BioLegend; anti-arginase-1 (A1exF5), Anti-CD8a (53-6.7), Anti-CD40 (1C10), anti-CD80 (16-10A1), anti-Foxp3 (clone FJK-16s), anti-granzyme B(NGZB), anti-interferonγ (IFNγ, XMG1.2), anti-iNOS (W16030C), anti-perforin (eBioOMAK-D), was obtained from eBioscience. Dead cells were gated out from these analyses using Fixable Viability Dye (Thermo Fisher Scientific). For intracellular staining (e.g., INFγ, arginase-1), cells were stimulated with eBioscience™ Cell Stimulation Cocktail plus protein transport inhibitors (catalog number: 00-4975-93, Invitrogen) at 37 °C for 6 hours before staining. For intracellular and intranuclear staining, cells were fixed and permeabilized with Intracellular Fixation & Permeabilization Buffer Set (Thermo Fisher Scientific). The stained cells were analyzed via flow cytometry (FACSLyric, BD Biosciences) and Flowjo software (BD Biosciences). Cell types were determined as follows; CD8+ T cells: CD45+/ CD3+/ CD8+, helper T cells: CD45+/ CD3+/ CD4+/Foxp3-, Tregs: CD45+/ CD3+/ CD4+/Foxp3-, B cells: CD45+/CD3-/CD19+, NK cells: CD45+/ CD3-/ NK1.1+, DCs: CD45+/ (F4/80)-/CD11c+/(I-A/I-E)+, PMN-MDSCs: CD45+/CD3-/CD11b+/Gr-1^hi^/Ly6C^int^, M-MDSCs: CD45+/CD3-/CD11b+/Gr-1^int^/Ly6C^hi^, macrophage: CD45+/CD11b+/(F4/80)+, cancer cells: CD45-/CD31-/CD140a-/EpCAM+, fibroblasts: CD45-/CD31-/CD140a+/EpCAM-, endothelial cells: CD45-/CD31+/CD140a-/EpCAM-.

**Statistical analysis**

Quantitative data were expressed as means ± SEM. The unpaired t test was used for two-group comparisons, and a one-way analysis of variance (ANOVA) followed by the Tukey test was employed for multi-group comparisons. For comparison of tumor volumes resulting from *in vivo* experiments, repeated-measures two-way ANOVA followed by the Tukey test was used for multi-group comparisons (Sidak’s test for two-group comparisons). The survival curves were calculated using the Kaplan-Meier method, with the log-rank test with Bonferroni correction used to compare between subgroups. All statistical analysis was performed with GraphPad Prism version 8 (GraphPad software, La Jolla, CA, USA). A *P* value of < 0.05 was considered statistically significant.

**3. Supplementary Figures**


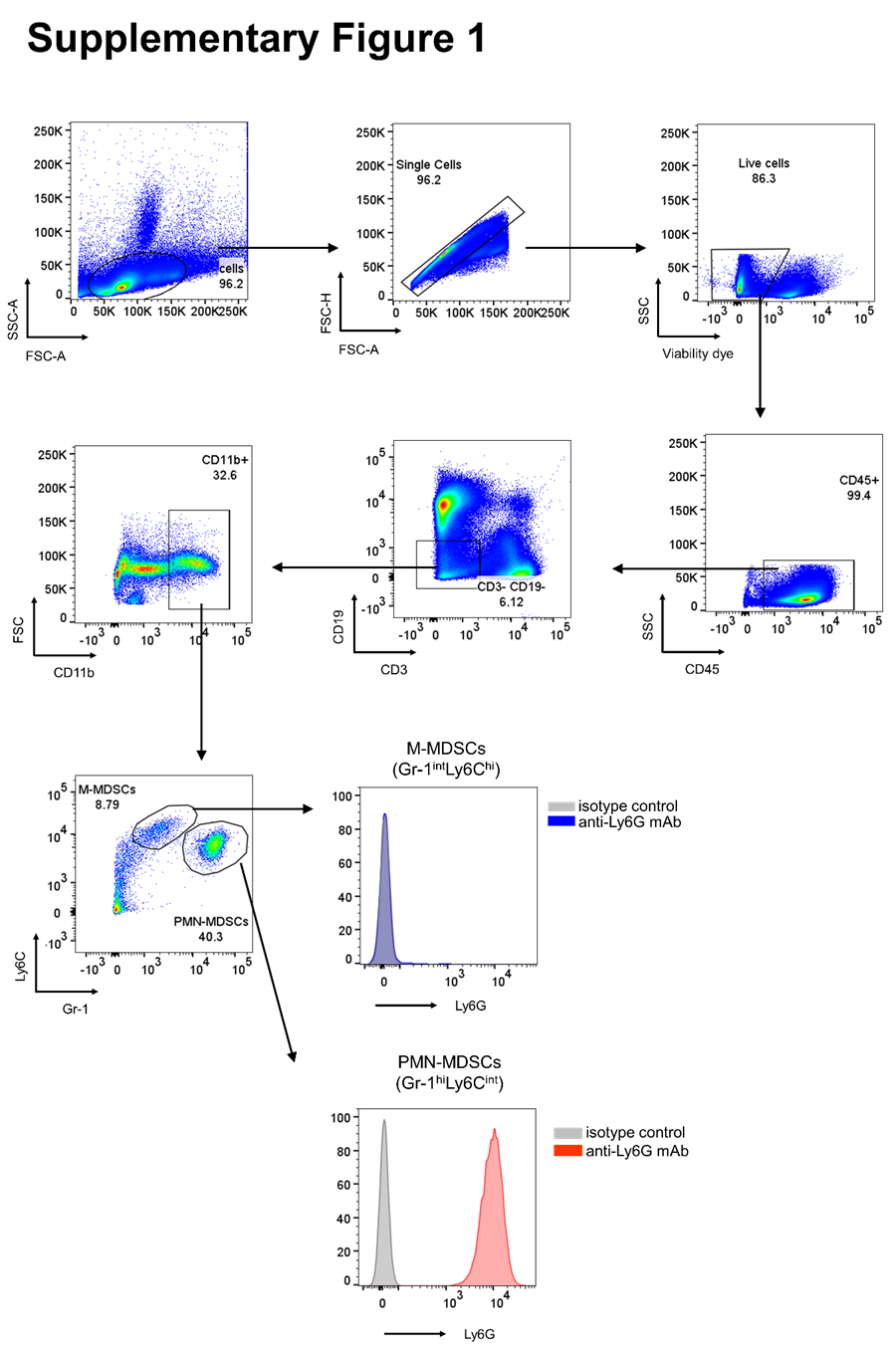

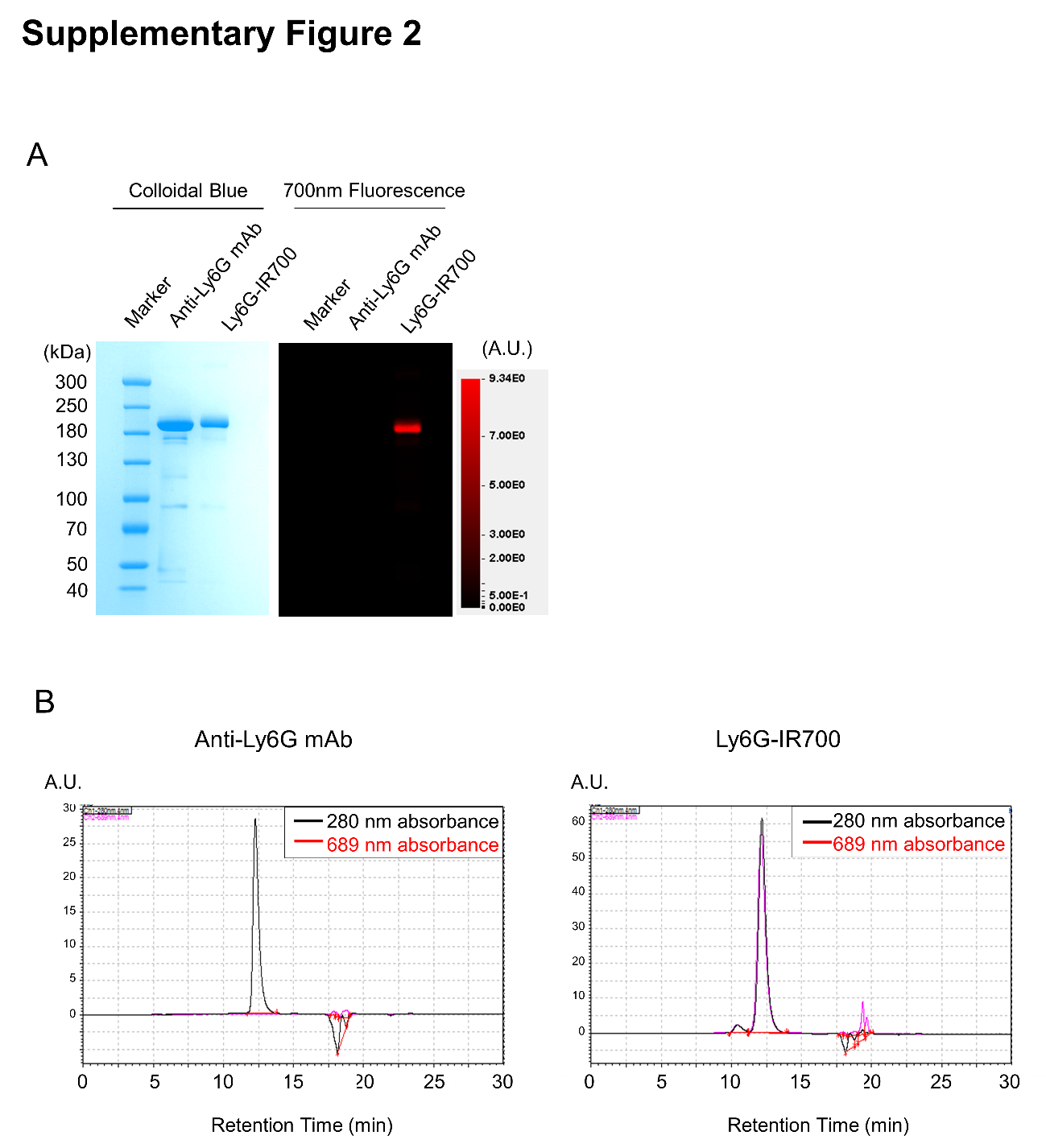


**Supplementary Figure S1.**

**Verification of the conjugation of anti-Ly6G-mAb to IR700.**

(A) Sodium dodecyl sulfate polyacrylamide gel electrophoresis (SDS-PAGE) with a 4–12 % polyacrylamide gel (Life Technologies, Gaithersburg, MD, USA) was used to assess the quality of APC. Non-conjugated antibody was used for the control. The gel was imaged by a Pearl Imager (LI-COR Biosciences, Lincoln, NE, USA) utilizing the 700 nm fluorescence channel after 2.5 hours of electrophoresis at 80 V. Then, the gel was dyed with colloidal blue to compare the conjugate's molecular weight to that of the non-conjugated antibody. (B) Size exclusion chromatography (SEC) was also used to test Ly6G-IR700. A Nexera XR UHPLC system (Shimadzu Co., Kyoto, Japan) was used for SEC analysis. Approximately 5 μg of protein was loaded onto a TSKgel SuperSW 3000 (4.6 mm x 30 cm, 5 m) column (Tosoh Bioscience, Inc., South San Francisco, CA, USA) and eluted with an isocratic flow of 200 mM sodium phosphate with 10% acetonitrile at pH 6.8 (30 min, 0.25 mL/min). The absorption of the elute was monitored at a wavelength of 280 and 689 nm. Verification of Ly6G-IR700 by SDS-PAGE (A, left: Colloidal Blue staining image, right: 700nm fluorescence image) and size exclusion chromatography (B, black: 280 nm absorbance, red: 689 nm absorbance). An unconjugated anti-Ly6G antibody was used as a control. A.U., arbitrary unit.


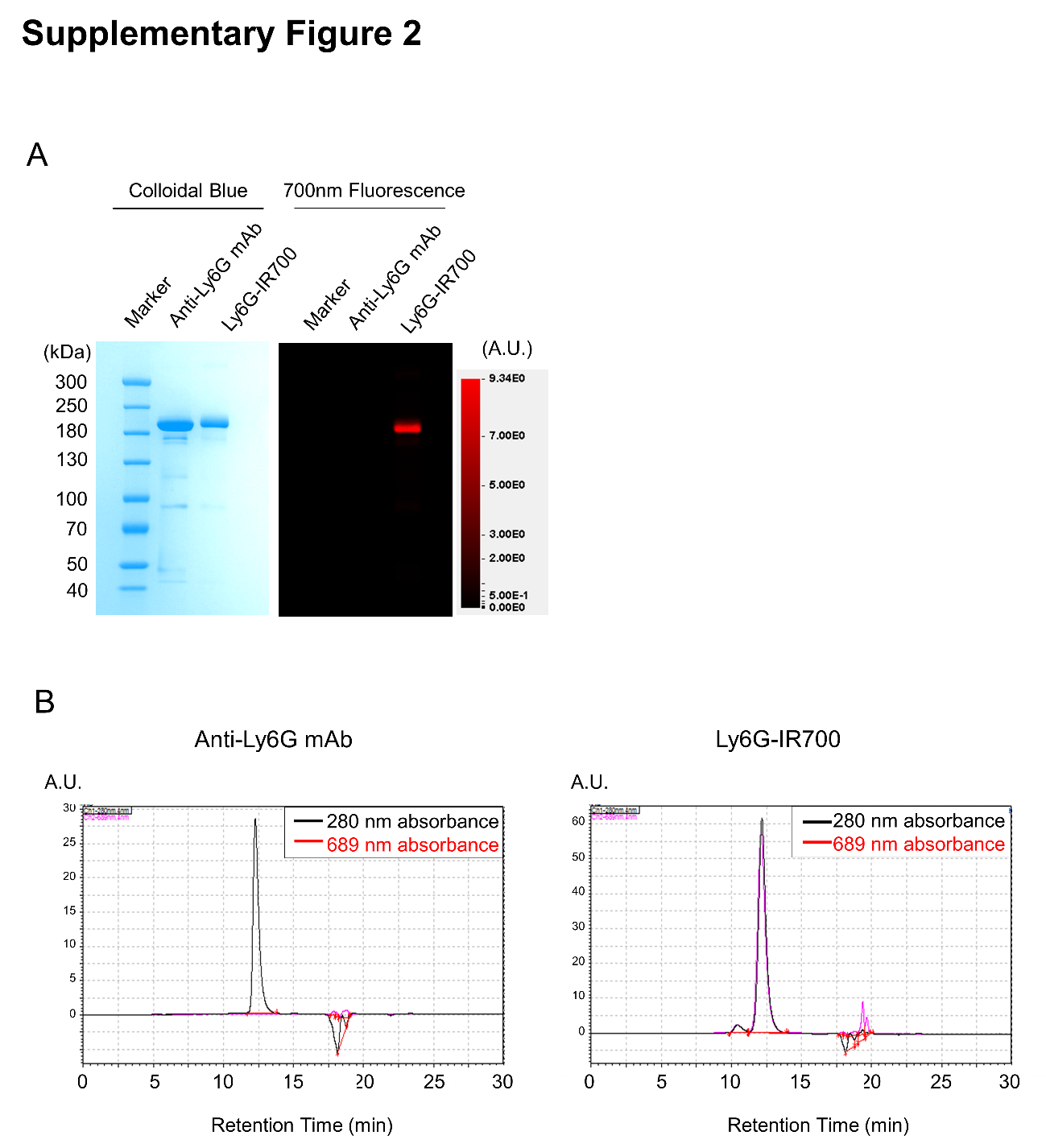


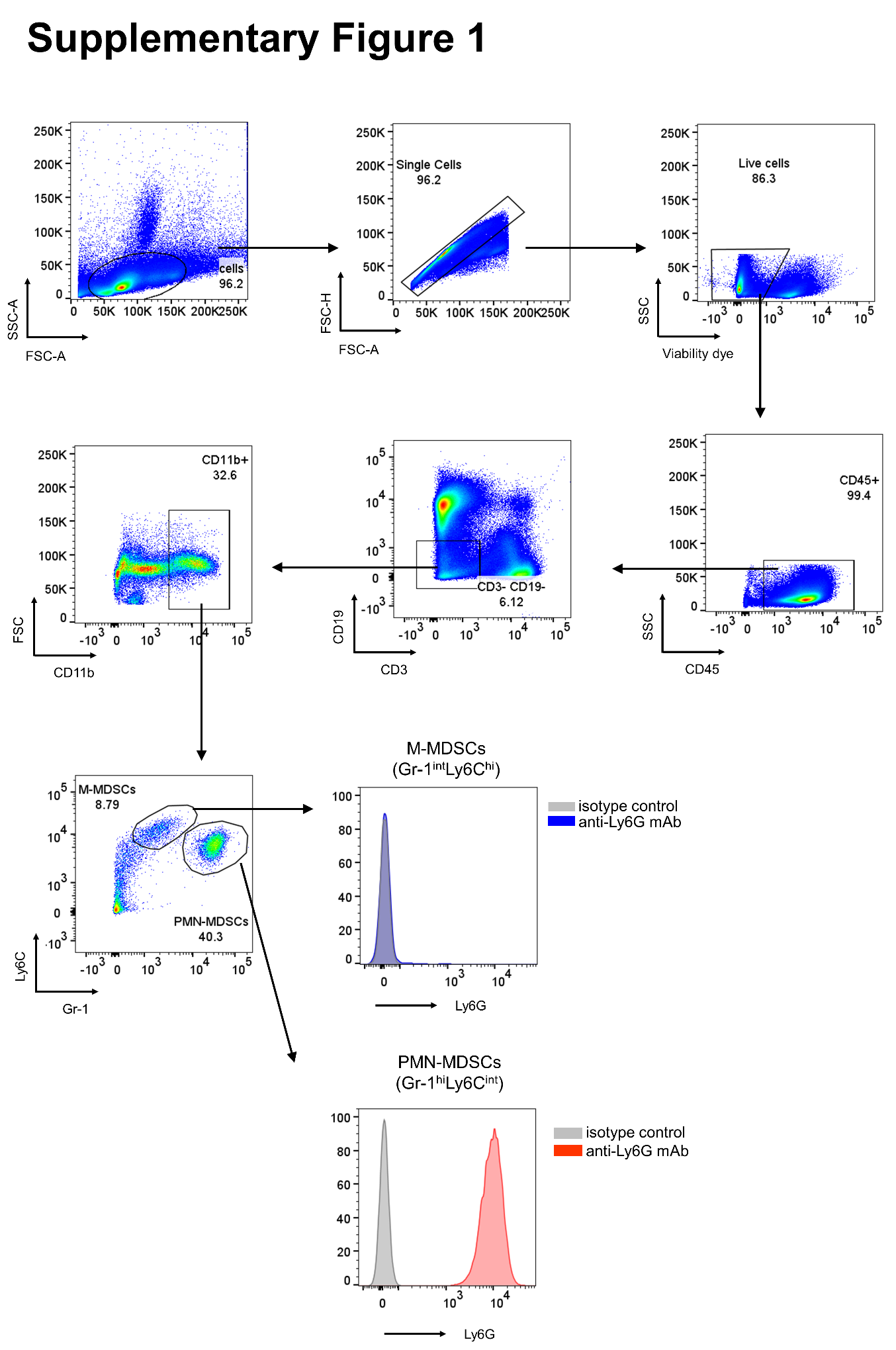


**Supplementary Figure S2.**

**Gating strategy and evaluation of Ly6G expressions for MDSCs using Gr-1 and Ly6C antibodies.**

Gating strategy and representative flow cytometry plots. Numbers in dot plots indicate frequencies of gated population (%) of parent population.

**Supplementary Figure S3**


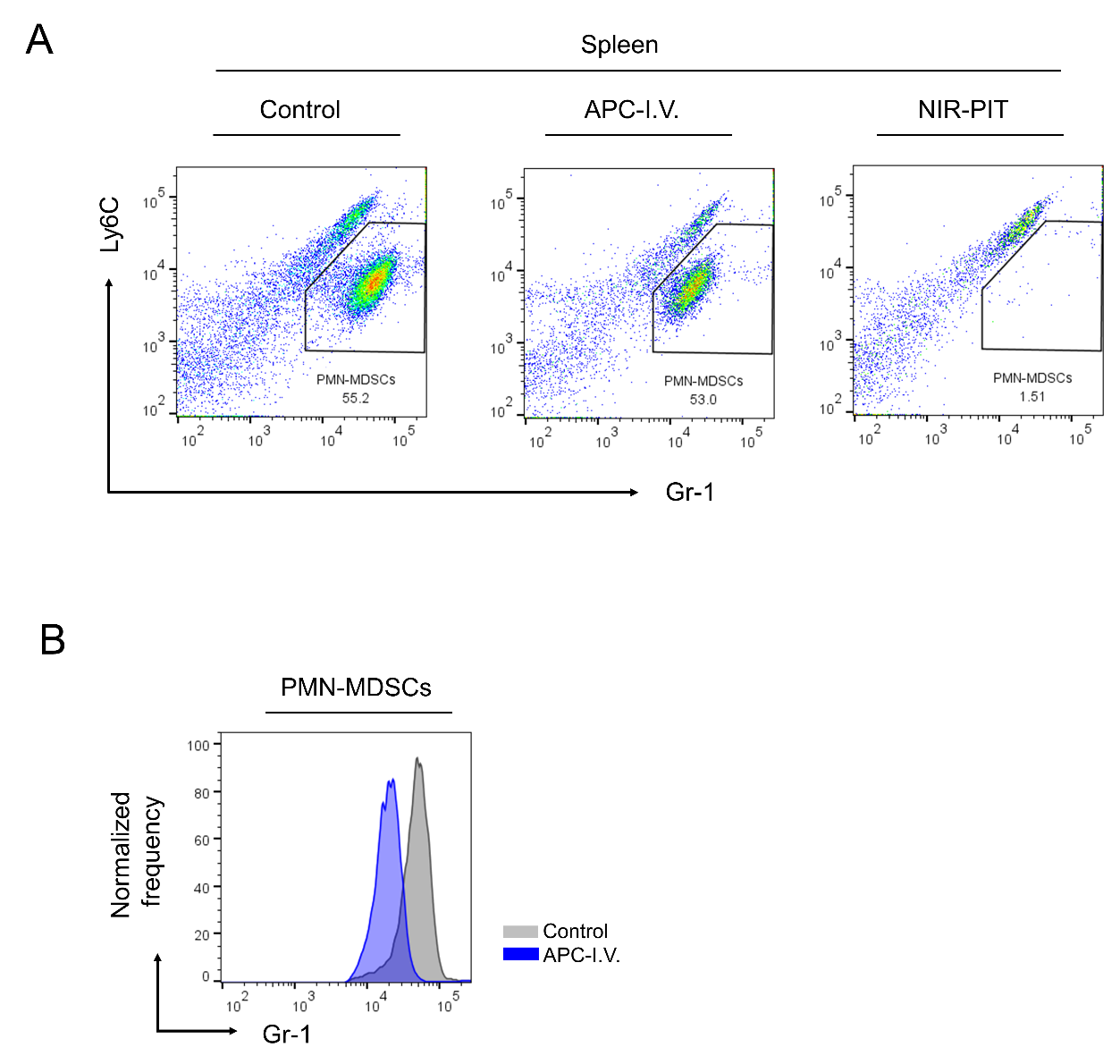


**Supplementary Figure S3.**

**Gating examples with Gr-1 and Ly6G-IR700.**

Representative gating examples in Figure 1C and 1D are shown. (A) Gating strategy of PMN-MDSCs using Gr-1 and Ly6C antibodies by dot plots. (B) Histograms of Gr-1 expression for examples of PMN-MDSCs in splenocytes. Although Ly6G-IR700 and Gr-1 antibody are mildly competitive, it is possible to gate PMN-MDSCs using Gr-1 and Ly6C antibodies.

**Supplementary Figure S4**

**
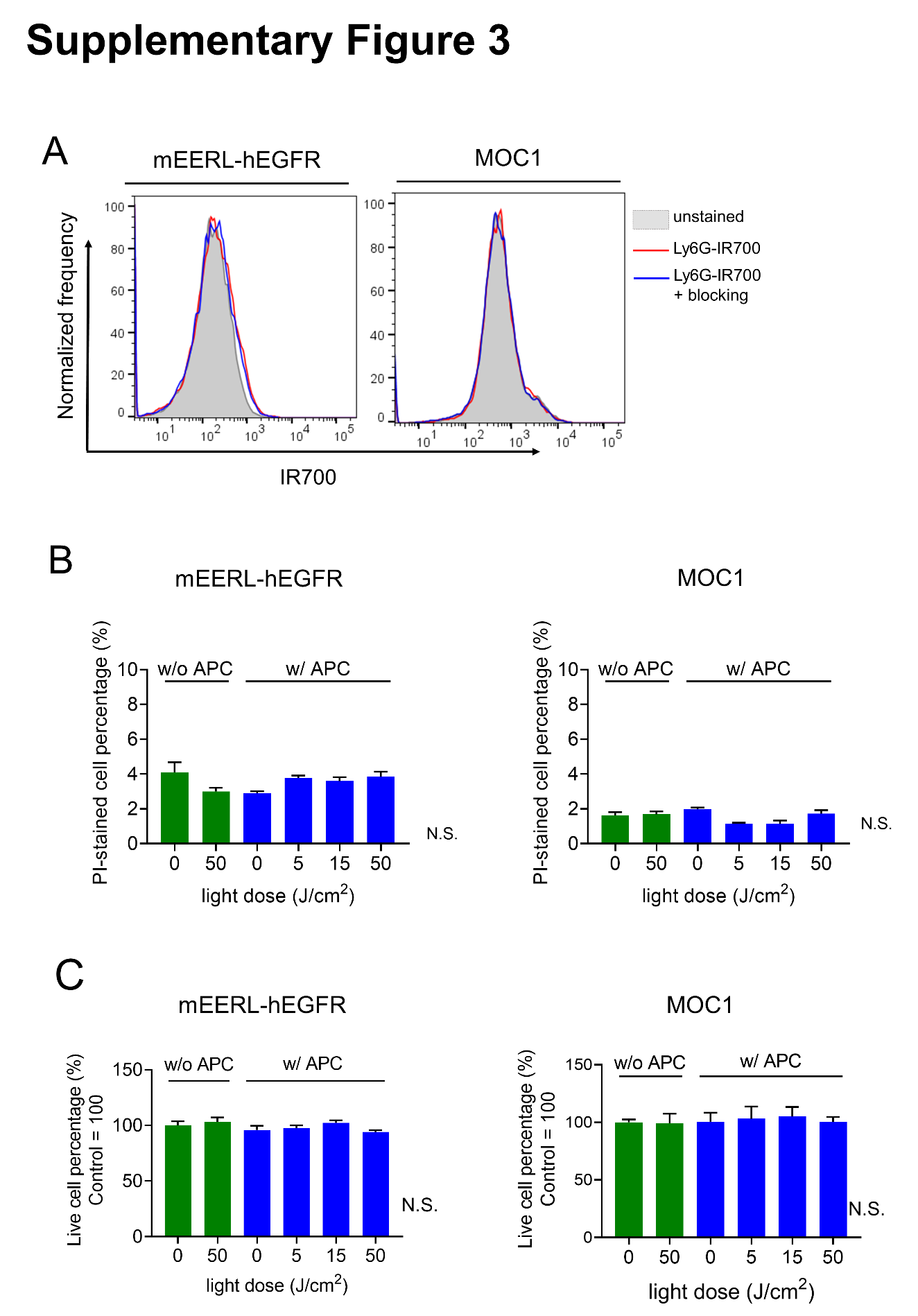
**

**Supplementary Figure S4.**

**The efficacy of Ly6G-targeted NIR-PIT against cancer cells.**

mEERL-hEGFR and MOC1 cells (2 × 10^5^) were seeded into 12-well plates, incubated for 24 hours, and then exposed to media containing Ly6G-IR700 (10 μg/mL) for 1 hours at 37 °C. After washing with PBS, phenol-red-free medium was added. Adequate NIR light was irradiated to cancer cells with an ML7710 laser system (Modulight, Tampere, Finland) at a power density of 150 mW/cm^2^. One hour after NIR-PIT, the cells were collected with trypsin, and stained with propidium iodide (PI, 1 µg/mL) at room temperature for 5 minutes, and then assessed for PI positivity on a BD FACSLyric (BD Biosciences). To assess metabolic activity, cell proliferation was evaluated by 3-(4,5-Dimethyl-2-thiazolyl)-2,5-diphenyl-2H-tetrazolium bromide (MTT) assay. Cells were incubated and treated, as described above. One hour after NIR-PIT, the medium was removed and 0.5 mg/mL of MTT reagent (SIGMA Aldrich) was added to each well. After 1 hour incubation, the supernatant was removed, and 500 μL of 2-propanol was added to each well to dissolve the crystal formazan dye. After transferring 100 μL of the supernatant to 96 well plate each, absorbance was measured at 570 nm on a microplate reader (SynergyTM H1). For relative quantification, the value of absorbance in each group was normalized to that in the control group. (A) Flow cytometric analysis of Ly6G-IR700 binding to mEERL-hEGFR and MOC1 cells. (B) The efficacy of Ly6G-targeted NIR-PIT against cancer cells was evaluated using PI staining by flow cytometry. (C) Metabolic activity by MTT assay. (B and C, Control was set as the group without APC administration or NIR light irradiation. n =5, one-way ANOVA followed by Dunnett’s test, means ± SEM, N.S., not significant).

**Supplementary Figure S5**


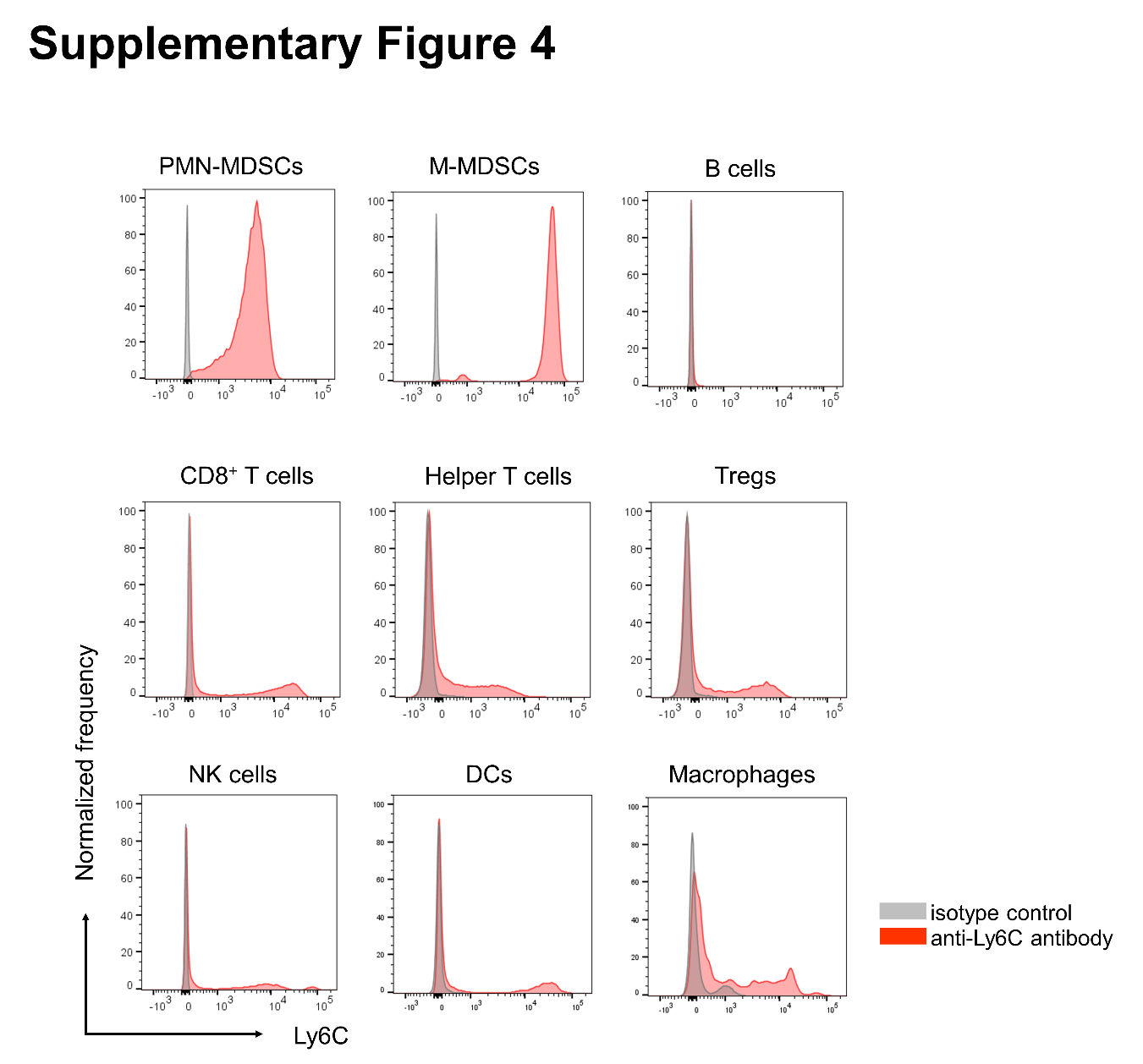


**Supplementary Figure S5.**

**Ly6C expression on various hematopoietic cells in spleen.**

Surface expressions of Ly6C on various hematopoietic cells were evaluated with flowcytometry. Ly 6G was expressed on not only M-MDSCs but also PMN-MDSCs, CD8^+^ T cells, helper T cells, Tregs, NK cells, DCs, and macrophages

**Supplementary Figure S6**


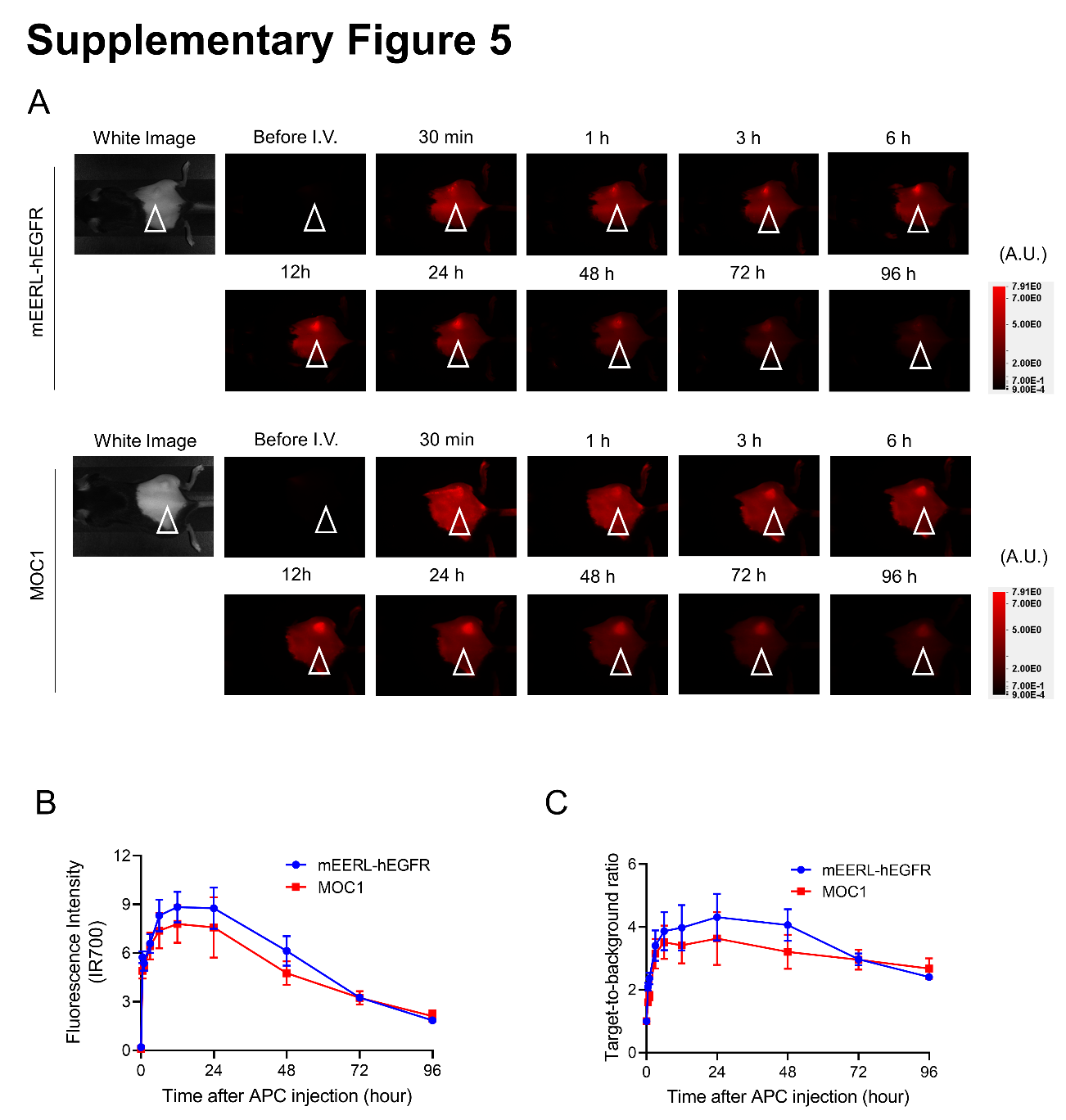


**Supplementary Figure S6.**

**In vivo IR700 fluorescence imaging of mEERL-hEGFR and MOC1 tumor.**

*In vivo*, IR700 fluorescence and white light images were obtained using a Pearl Imager (700nm fluorescence channel; LI-COR Biosciences) and analyzed using Pearl Cam Software (LI-COR Biosciences). Serial dorsal fluorescence images of the IR700 signal were obtained before and 1/2, 1, 3, 6, 12, 24, 48, 72, and 96 hours after intravenous injection of 100 mg of Ly6G-IR700 via tail vein. ROIs were placed on the tumor and the adjacent nontumor region (left dorsum) as background. The mean value of fluorescence intensity was calculated for each ROI. Target-to-background ratio (TBR) was calculated from fluorescence intensity of tumor and fluorescence intensity of background by the following formula: (mean fluorescence intensity of tumor)/(mean fluorescence intensity of background). (A) In vivo Ly6G–IR700 fluorescence real-time imaging of mEERL-hEGFR or MOC1-bearing mice. The tumor showed high fluorescence intensity after injection and the intensity was gradually decreased over days. Arrowhead shows the tumor (A.U., arbitrary unit). (B) Quantitative analyses of mean fluorescence intensity in both tumors are shown (n = 5). (C) Quantitative analysis of TBR in both tumors (n = 5).

**Supplementary Figure S7**


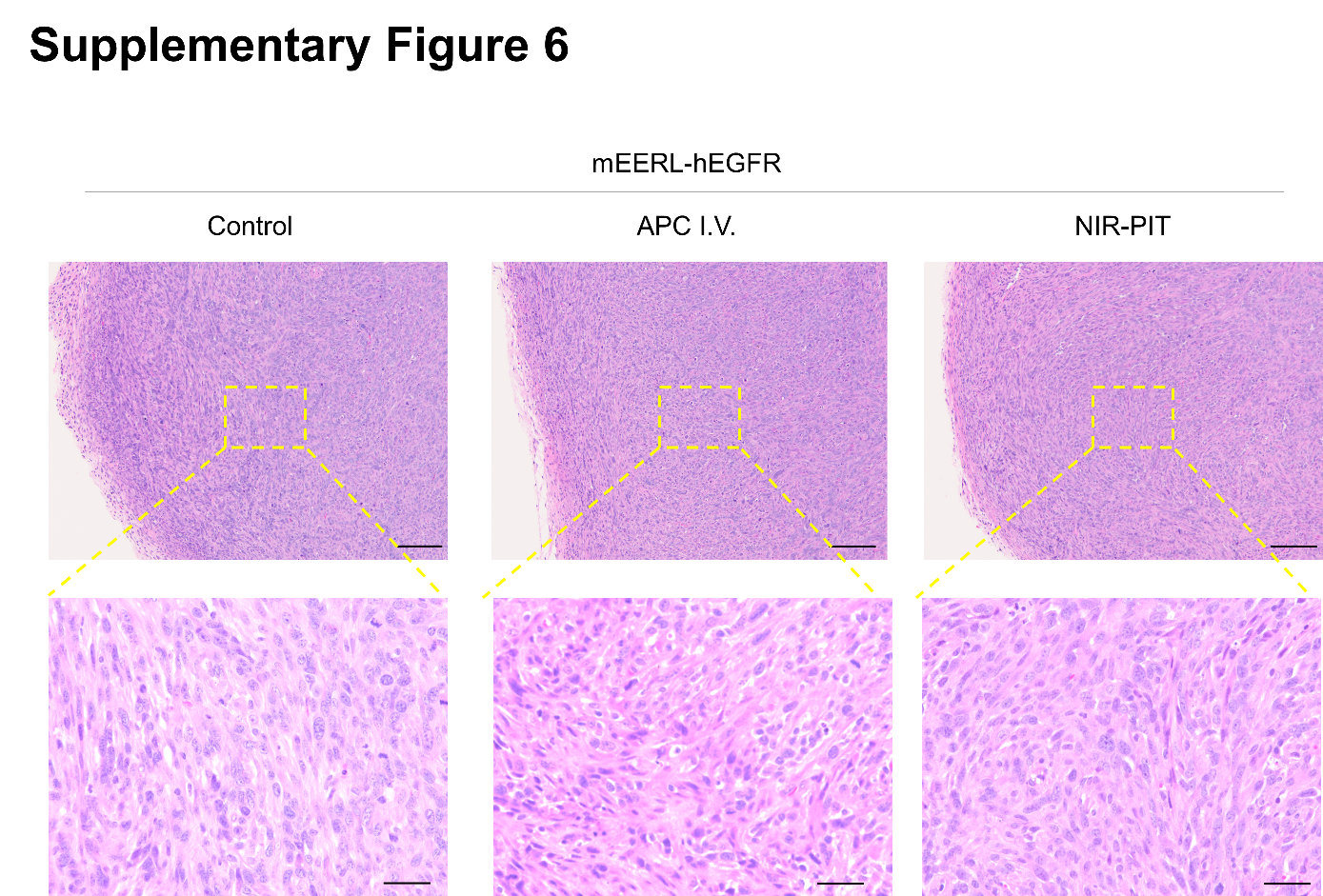


**Supplementary Figure S7.**

**Histological changes after Ly6G-targeted NIR-PIT in mEERL-hEGFR tumor bearing mice.**

Tumors from mEERL-hEGFR model were resected, formalin-fixed and paraffin-embedded, and sectioned. Hematoxylin and eosin staining, bright-light images were acquired using Mantra Quantitative Pathology Workstation (Akoya Biosciences, Menlo Park, CA, USA). No obvious morphological changes were found. Lower figures are magnified images. Scale bar, 100 µm (top) and 50 µm (bottom).

**Supplementary Figure S8**


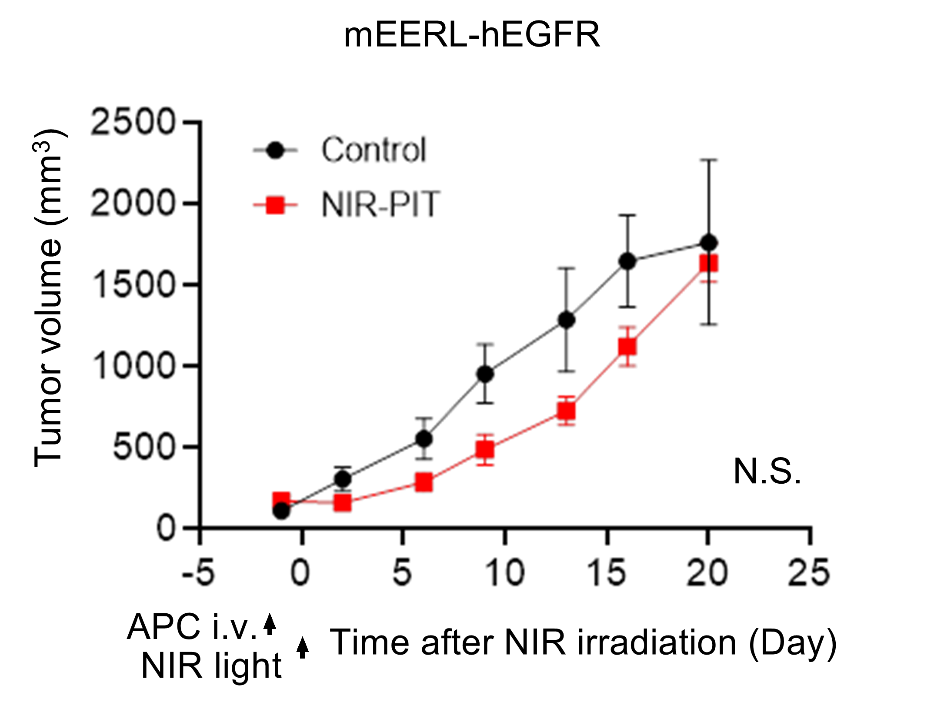


**Supplementary Figure S8.**

**The efficacy of in vivo Ly6G-targeted NIR-PIT in athymic mice.**

Six to eight-week-old female homozygote athymic nude mice were purchased from Charles River (Frederick, MD, USA). Tumors were established via subcutaneous injection of 1 × 10^6^ cells for mEERL-hEGFR tumor in the right dorsal flank. For Ly6G-targeted NIR-PIT experiments, tumor-bearing mice were randomized into two groups as follows: (i) no treatment (control), (ii) intravenous injection of Ly6G-IR700 (100 μg) followed by NIR light irradiation (NIR-PIT). 24 hours after Ly6G-IR700 administration, NIR light (690 nm, 150 mW/cm^2^, 50 J/cm^2^) was applied to the tumors. Tumor volume curve is shown (n = 5; mean ± SEM; repeated measures two-way ANOVA followed by Sidak’s test; N.S., not significant).

**Supplementary Figure S9**


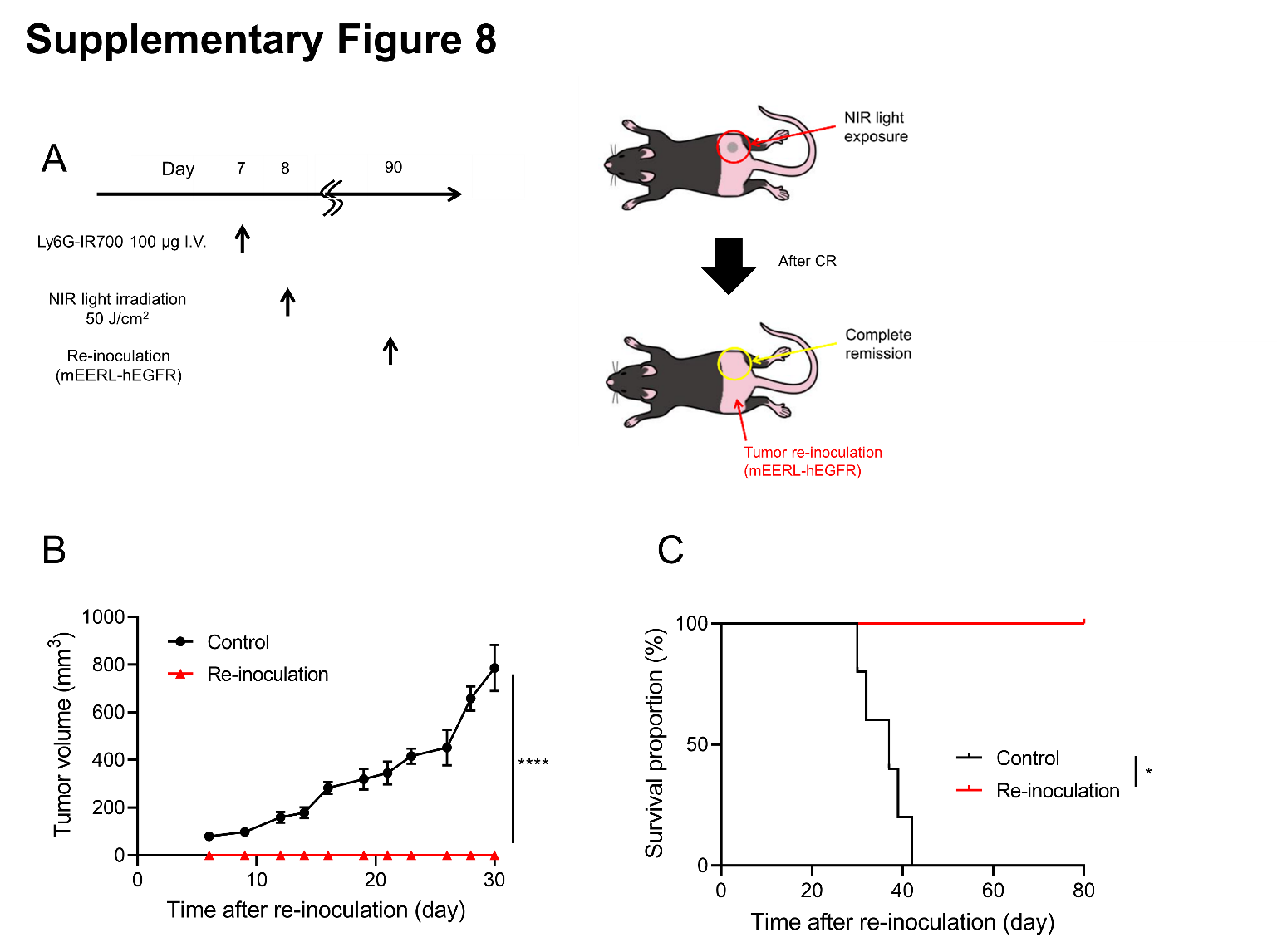


**Supplementary Figure S9.**

**Re-inoculation of cancer cells after Ly6G-targeted NIR-PIT.**

The mice that achieved complete remission by Ly6G-targeted NIR-PIT were re-inoculated with mEERL-hEGFR cells in the contralateral side. (A) Treatment schedule and diagram of re-inoculation. The red circle indicates NIR light irradiation site for the initial treatment. The yellow circle shows the cleared tumor. The red arrow points to the re-inoculation site. (B) Tumor volume curves. (n = 3; mean ± SEM; repeated measures two-way ANOVA followed by Sidak’s test; ****, P < 0.0001). (C) Survival curves. (n = 3; log-rank test; *, P < 0.05).

**Supplementary Figure S10**


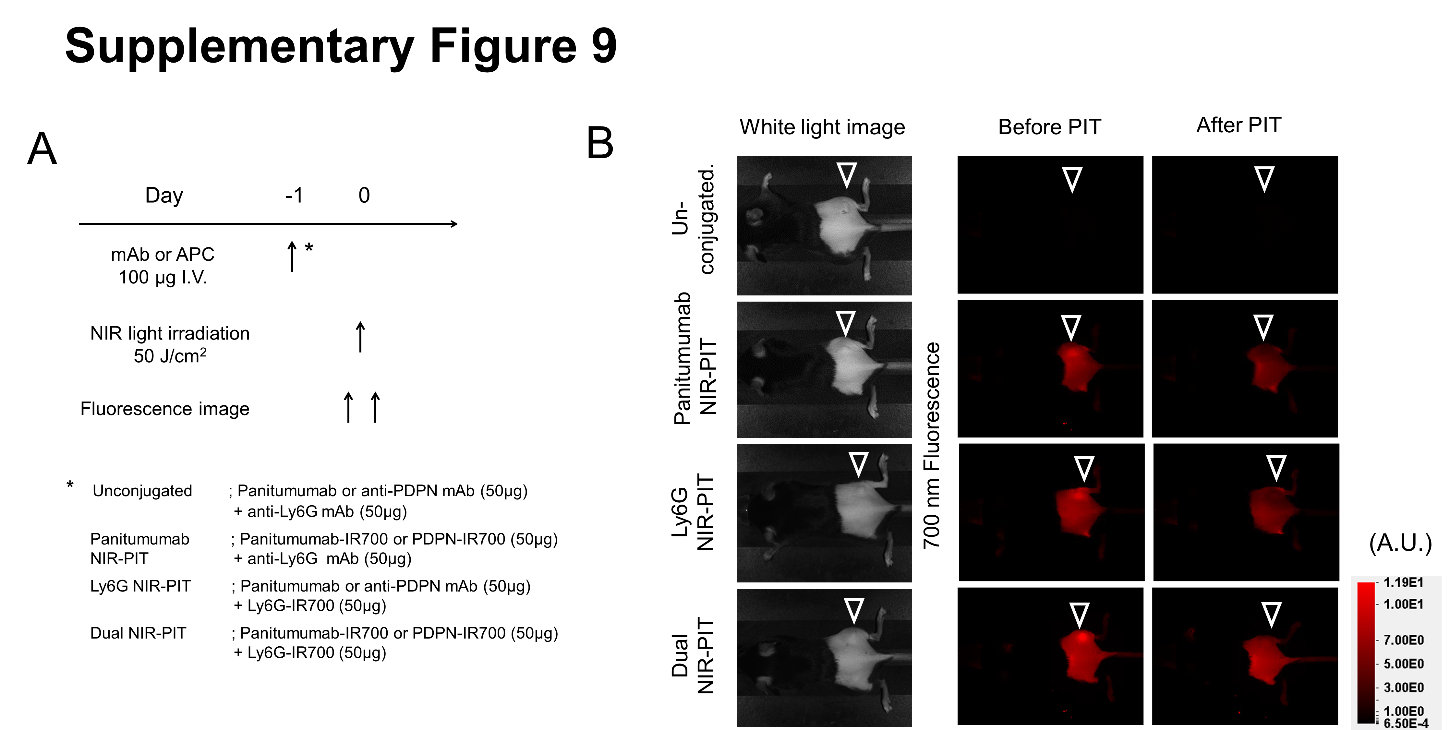


**Supplementary Figure S10.**

**Treatment regimen and 700 nm fluorescence images for combined NIR-PIT.**

(A) Treatment schedule are presented. (B) Fluorescent images of 700 nm before and after NIR-PIT in mEERL-hEGFR tumor-bearing mice. Arrowheads show tumors (A.U.; arbitrary units).
